# Distinct mRNA expression profiles and miRNA regulators of the PI3K/AKT/mTOR pathway in breast cancer: insights into tumor progression and therapeutic targets

**DOI:** 10.3389/fonc.2024.1515387

**Published:** 2025-01-09

**Authors:** Tomasz Sirek, Katarzyna Król-Jatręga, Przemysław Borawski, Nikola Zmarzły, Dariusz Boroń, Piotr Ossowski, Olga Nowotny-Czupryna, Kacper Boroń, Dominika Janiszewska-Bil, Elżbieta Mitka-Krysiak, Beniamin Oskar Grabarek

**Affiliations:** ^1^ Department of Plastic Surgery, Faculty of Medicine, Academia of Silesia, Katowice, Poland; ^2^ Department of Plastic and Reconstructive Surgery, Hospital for Minimally Invasive and Reconstructive Surgery in Bielsko-Biała, Bielsko-Biala, Poland; ^3^ Independent Researcher, Włocławek, Poland; ^4^ Department of Medical and Health Sciences, Collegium Medicum, WSB University, Dabrowa Górnicza, Poland; ^5^ Department of Gynecology and Obstetrics with Gynecologic Oncology, Ludwik Rydygier Memorial Specialized Hospital, Kraków, Poland; ^6^ Department of Gynecology and Obstetrics, TOMMED Specjalisci od Zdrowia, Katowice, Poland; ^7^ University of Economics and Humanities in Warsaw, Warszawa, Poland; ^8^ Department of Molecular, Biology Gyncentrum Fertility Clinic, Katowice, Poland

**Keywords:** breast cancer, miRNA, PI3K/Akt/mTOR pathway, molecular marker, mRNA

## Abstract

**Background:**

Breast cancer remains a leading cause of mortality among women, driven by the molecular complexity of its various subtypes. This study aimed to investigate the differential expression of genes and miRNAs involved in the PI3K/AKT/mTOR signaling pathway, a critical regulator of cancer progression.

**Methods:**

We analyzed tumor tissues from five breast cancer subtypes—luminal A, luminal B HER2-negative, luminal B HER2-positive, HER2-positive, and triple-negative breast cancer (TNBC)—and compared them with non-cancerous tissues. Microarray and qRT-PCR techniques were employed to profile mRNAs and miRNAs, while bioinformatic tools predicted miRNA-mRNA interactions. Statistical analysis was performed with a statistical significance threshold (p) < 0.05.

**Results:**

We identified several upregulated genes across all subtypes, with TNBC and HER2-positive cancers showing the most significant changes. Key genes such as *COL1A1*, *COL4A1*, *PIK3CA*, *PIK3R1*, and *mTOR* were found to be overexpressed, correlating with increased cancer aggressiveness. miRNA analysis revealed that miR-190a-3p, miR-4729, and miR-19a-3p potentially regulate these genes, influencing the PI3K/AKT/mTOR pathway. For instance, reduced expression of miR-190a-3p may contribute to the overexpression of PIK3CA and other pathway components, enhancing metastatic potential.

**Conclusion:**

Our findings suggest that the PI3K/AKT/mTOR pathway and its miRNA regulators play crucial roles in breast cancer progression, particularly in aggressive subtypes like TNBC. The identified miRNAs and mRNAs hold potential as biomarkers for diagnosis and treatment, but further validation in functional studies is required. This study provides a foundation for targeted therapies aimed at modulating this critical pathway to improve breast cancer outcomes.

## 1 Introduction

Breast cancer remains the most commonly diagnosed malignancy among women globally, as reported by the World Health Organization (WHO) in 2020 (1). In Poland, cancer is the second leading cause of death, with malignant tumors being the primary cause of mortality among women under the age of 65 (2). This emphasizes the critical need for improved diagnostic and therapeutic strategies (2).

Breast cancer is a highly heterogeneous disease, encompassing various molecular and clinicopathological features (3–5). Based on differences in the expression of key receptors such as estrogen receptor (ER), progesterone receptor (PgR), and human epidermal growth factor receptor 2 (HER2), as well as the proliferation marker Ki67, breast cancer is typically classified into four major subtypes: luminal A, luminal B, HER2-positive (HER2+), and triple-negative breast cancer (TNBC) (6–10). Each subtype carries distinct prognostic outcomes and necessitates tailored therapeutic approaches (3–5).

The luminal A subtype, known for its ER and PgR positivity and low Ki67 levels, generally exhibits a favorable prognosis and responds well to hormonal therapies (6–10). In contrast, luminal B can be further subdivided into HER2-negative and HER2-positive categories, both expressing ER but differing in HER2 status and proliferation rates. HER2-positive breast cancer, characterized by the overexpression of the HER2 protein, drives aggressive tumor growth and typically requires anti-HER2 therapies (6–10). TNBC, lacking ER, PgR, and HER2 expression, is recognized for its aggressive progression and poorer outcomes, often necessitating chemotherapy and other targeted treatments (6–10).

Breast cancer was one of the first malignancies where treatment protocols were tailored based on molecular profiling (11). Antiestrogen therapies, for instance, marked an early breakthrough in precision medicine by utilizing the ER status of tumors to guide treatment (12–16). The identification of HER2 overexpression, present in about 25% of breast cancers, further revolutionized treatment options with the development of effective anti-HER2 therapies such as trastuzumab and pertuzumab (12–16). Together, the ER, PR, and HER2 status form the foundation for treatment decisions in modern breast cancer management, with additional biomarkers like BRCA1/2 mutations and PD-1 further informing therapeutic strategies (12–16). While this system has significantly advanced individualized care, the inherent heterogeneity of breast cancer has necessitated further molecular characterization (12–16).

One critical pathway implicated in breast cancer progression is the PI3K/AKT/mTOR signaling cascade, which plays a pivotal role in regulating cellular processes such as metabolism, growth, proliferation, apoptosis, and angiogenesis (17). Class I PI3K consists of four catalytic subunits—p110α, p110β, p110γ, and p110δ—that interact with regulatory subunits, primarily of the p85 type. While p110α and p110β are broadly expressed, p110δ and p110γ are more restricted to hematopoietic cells. Dysregulation of the PI3K pathway, particularly within the class IA PI3K, has been implicated in various human diseases, including cancer, with mutations leading to altered enzymatic activity that drives oncogenesis (18–20).

In breast cancer, dysregulation of the PI3K/AKT/mTOR pathway occurs through several mechanisms, such as increased PI3K activity, loss of inhibitory functions, or mutations in tumor suppressor genes like INPP4B and PTEN. Among the PI3K genes, PIK3CA is the most frequently mutated, with alterations occurring predominantly in two hotspot regions: the helical domain (E542, E545, Q546) and the kinase domain (H1047). These mutations can either reduce p85-mediated inhibition or directly enhance the lipid kinase activity of the p110α catalytic subunit, thus promoting tumor growth (21–23).

PIK3CA mutations are particularly common in early breast cancer, present in up to 47% of hormone receptor-positive (HR+)/HER2– luminal A tumors, 33% of HR+/HER2+ luminal B tumors, 39% of HER2-enriched tumors, and 8–25% of basal-like or triple-negative tumors (22, 24, 25). Such mutations are also found in metastatic breast cancer, indicating their role in both early and advanced disease stages (24, 25). Amplification of the PIK3CA gene locus and other rare genetic alterations, such as PIK3CB amplification or PIK3R1 inactivating mutations, have been identified, further highlighting the complexity of this pathway’s involvement in breast cancer (24, 25).

Additionally, microRNAs (miRNAs) have emerged as key players in breast cancer development by regulating gene expression at the post-transcriptional level. These small, non-coding RNAs contribute to the intricate molecular landscape of tumors by modulating the expression of oncogenes and tumor suppressors (26, 27), including those involved in the PI3K/AKT/mTOR pathway, thereby adding another layer of complexity to cancer biology (28).

Moreover, miRNAs play a crucial role in regulating key signaling pathways involved in breast cancer development, including those associated with hormone receptor status, HER2/neu overexpression, and resistance to both chemotherapy and targeted therapies (29). Specific miRNAs have been identified that modulate the expression of critical receptors such as estrogen receptor (ER), progesterone receptor (PR), and HER2, directly influencing tumor progression and treatment response (30, 31). These regulatory functions of miRNAs highlight their potential as both biomarkers and therapeutic targets in breast cancer (30, 31).

Therefore, this study aimed to assess the expression profile of mRNA and miRNA related to the PI3K/AKT/mTOR signaling cascade in five types of breast cancer in Polish women.

## 2 Materials and methods

### 2.1 Ethics

This study followed ethical guidelines based on the 2013 Declaration of Helsinki for human research. Approval was granted by the Bioethical Committee of the Regional Medical Chamber in Krakow (Ref: 81/KBL/OIL/2023, March 10, 2023). Data confidentiality and patient anonymity were strictly maintained, with all identifying information removed from the database before analysis, ensuring patient identities remained anonymous.

### 2.2 Subjects

The study included patients with five breast cancer subtypes: luminal A (n = 130), luminal B [n = 196, divided into HER2-negative (n = 100) and HER2-positive (n = 96)], HER2-positive (n = 36), and triple-negative breast cancer (TNBC) (n = 43). During surgery, tumor tissue and healthy tissue (control) were excised. Pathomorphological evaluation determined if healthy margins were obtained, expanding surgery if necessary. Tumor-affected tissue was compared with non-affected control tissue based on intraoperative assessments. Notably, all patients were classified according to the Tumor, Nodules, and Metastases (TNM) classification system as T1N0M0 (32). Detailed patient data is available in **Table 1**.

**Table 1:** Patient characteristics.

Molecular Type	Comorbidities	G1	G2	G3	Age < 50 years	Age > 50 years/BMI [kg/m2]
Luminal A		23 (18%)	48 (37%)	59 (45%)	43 (33%)	87 (67%)/30.78 ± 2.76
	Hypertension	3 (13%)	6 (13%)	7 (12%)	5 (12%)	11 (13%)
	Diabetes mellitus type 2	2 (9%)	5 (10%)	3 (6%)	3 (7%)	7 (8%)
	Hypertension + Diabetes type 2	3 (13%)	3 (6%)	2 (3%)	2 (5%)	6 (7%)
	None	15 (65%)	34 (71%)	47 (79%)	33 (77%)	63 (72%)
Luminal B HER2-		31 (31%)	57 (57%)	12 (12%)	32 (32%)	68 (68%)/30.18 ± 4.56
	Hypertension	4 (13%)	8 (14%)	1 (8%)	5 (16%)	8 (12%)
	Diabetes mellitus type 2	3 (10%)	5 (9%)	1 (8%)	4 (13%)	5 (7%)
	Hypertension + Diabetes type 2	3 (10%)	4 (7%)	0 (0%)	3 (9%)	4 (6%)
	None	21 (68%)	40 (70%)	10 (83%)	20 (62%)	51 (75%)
Luminal B HER2+		23 (24%)	57 (59%)	16 (17%)	19 (20%)	77 (80%)/32.09 ± 6.19
	Hypertension	3 (13%)	7 (12%)	2 (12%)	3 (16%)	9 (12%)
	Diabetes mellitus type 2	2 (9%)	6 (11%)	1 (6%)	2 (11%)	7 (9%)
	Hypertension + Diabetes type 2	2 (9%)	4 (7%)	1 (6%)	2 (11%)	5 (6%)
	None	16 (69%)	40 (70%)	12 (75%)	12 (63%)	56 (73%)
Non-luminal HER2+		9 (25%)	12 (33%)	15 (42%)	9 (25%)	27 (75%)/33.18 ± 5.67
	Hypertension	1 (11%)	2 (17%)	2 (13%)	2 (22%)	3 (11%)
	Diabetes mellitus type 2	1 (11%)	1 (8%)	1 (7%)	1 (11%)	2 (7%)
	Hypertension + Diabetes type 2	1 (11%)	1 (8%)	1 (7%)	1 (11%)	2 (7%)
	None	6 (67%)	8 (67%)	11 (73%)	5 (56%)	20 (74%)
TNBC		14 (32%)	21 (49%)	8 (19%)	10 (23%)	33 (77%)/34.67 ± 2.98
	Hypertension	2 (14%)	3 (14%)	1 (12%)	2 (20%)	4 (12%)
	Diabetes mellitus type 2	1 (7%)	2 (10%)	0 (0%)	1 (10%)	2 (6%)
	Hypertension + Diabetes type 2	2 (14%)	2 (10%)	0 (0%)	1 (10%)	3 (9%)
	None	9 (64%)	14 (67%)	7 (88%)	6 (60%)	24 (73%)

Data are presented as number of cases and (percentage) or mean ± standard deviation; HER, human epidermal growth factor receptor 2; TNBC, triple-negative breast cancer; BMI, body mass index.

### 2.3 Isolation of total ribonucleic acid

Total RNA extraction from tissues was performed using TRIzol reagent (Invitrogen Life Technologies, California, USA, Catalog: 15596026) following the manufacturer’s instructions. The RNeasy mini kit (QIAGEN, Valencia, CA, USA, Catalog: 74104) was then used to purify the RNA, removing contaminants. DNase I treatment (Fermentas International Inc., Burlington, Canada, Catalog: 18047019) was applied to eliminate any genomic DNA. RNA quality was assessed via 1% agarose gel electrophoresis with ethidium bromide, and concentration was measured by absorbance at 260 nm to determine yield and purity.

### 2.4 Microarray profiling of PI3K/AKT/mTOR pathway genes

The list of genes associated with circadian clock-related genes was compiled using data from the Kyoto Encyclopedia of Genes and Genomes (KEGG database; http://pathcards.genecards.org/, accessed on May 4, 2024) (33). Gene expression analysis of PI3K/AKT/mTOR pathway genes in tumor versus control tissues was conducted using the HG-U 133_A2 microarray (Affymetrix, Catalog: 902416). Of 22,277 probes on the array, 390 targeted this pathway, identified through the NetAffx Analysis Center. The process included cDNA synthesis, RNA amplification, and hybridization, followed by fluorescence intensity measurement with the Affymetrix Gene Array Scanner 3000 7G.

### 2.5 Comprehensive microarray profiling of PI3K/AKT/mTOR pathway related miRNAs and their potential impact on gene expression

Microarray analysis of miRNAs involved in the PI3K/AKT/mTOR pathway was performed using the GeneChip miRNA 2.0 Array (Affymetrix, Santa Clara, CA). Differentially expressed miRNAs between tumor and control tissues were identified using the TargetScan (34) and miRanda (35) databases. Predicted miRNA-mRNA interactions with scores above 80 were deemed highly credible, while those below 60 required further validation (35, 36). This analysis aimed to understand the regulatory impact of miRNAs on gene expression within the pathway.

### 2.6 Quantitative reverse transcription polymerase chain reaction analysis

To validate the microarray results, qRT-PCR was performed using the SensiFast SYBR No-ROX One-Step kit (Bioline, London, UK) according to the manufacturer’s instructions. Gene expression was analyzed using the 2^−ΔΔCt^ method, with a fold change of 1 representing control levels, values above 1 indicating overexpression, and below 1 indicating silencing. β-actin (*ACTB*) was used for normalization, and primer sequences are listed in **Table 2**. All primers used in the study were purchased from Sigma-Aldrich (St. Louis, MO, USA), and the specificity of the RT-qPCR reactions was confirmed by the melting temperature (Tm) of the PCR products.

**Table 2:** Nucleotide sequence of primers.

mRNA	Nucleotide sequence	
*COL1A1*	Forward	5’- GAGGGCCAAGACGAAGACATC -3’
	Reverse	5’- CAGATCACGTCATCGCACAAC -3’
*COL2A1*	Forward	5’- TTGTTTTAAGGGTTTTTTGTATGAG-3’
	Reverse	5’- TATCTCCAAATTCTCCTTTCTATCC-3’
***COL4A1***	Forward	5’- GGGAGTATTTGGTTATTTTGGAAAT-3’
	Reverse	5’- AAATCACCCTTCATCCCTAATAAAC -3’
*COL4A4*	Forward	5’- ATTTAGGGAGATAGAGGGGATTTAG-3’
	Reverse	5’- AAAAAACCTTACTAACCAACCTCAC-3’
*COL4A6*	Forward	5’- GGATTAATTGGAAAAATGGGATTAT-3’
	Reverse	5’- ACCTCTCCTTTATCACCTCTTAACC-3’
*MAPK1*	Forward	5’- TGGAATAGGTTGTTTTTAAATGTTG-3’
	Reverse	5’- AAACTTTTCCTTAAACAAATCATCC-3’
*MAPK3*	Forward	5’- AAGATTAAGGTGGTTTGGGTTAAGT-3’
	Reverse	5’- TAAATAAATCATCCAACTCCATAAC-3’
*MAP2K2*	Forward	5’- TTTTAGAGGGGAGATTAAGTTGTGT-3’
	Reverse	5’- AAAACAAACCCATACTCCAAATATC-3’
*PIK3CG*	Forward	5′- GTGGTGTTGAGAGAGGATAATTGT-3 ′
	Reverse	5′-AACCACACCTAAACCTTCATCTACT-3 ′
*PIK3R1*	Forward	5’- GGTTGGGTAATGAAAATATTGAAGAT-3’
	Reverse	5’- TCCACCACTACAAAACAAACATAAC-3’
*PIK3R4*	Forward	5’- TGTTGGGGTTGTAGTAGTTGAAGT-3’
	Reverse	5’- CCCCTAACCTACCCTAAAAAACATA-3’
*mTOR*	Forward	5’- AAGGTTATTTTTTGATTTAATTTGTT-3’
	Reverse	5-’ ACACATCTACCACCACTTACACTAC-3’
*PIK3CA*	Forward	5’- TTGGTAAAATTAAGGTTTTGATTTT-3’
	Reverse	5’- TAAACTACAATACACCTTTCAAACC-3’
*PIK3C*B	Forward	5’- TTTGAGGATGTTGTTTAGTTTTAGG-3’
	Reverse	5’- AAAAAATTCTTCATCACTCATCTATC-3’
*PIK3CD*	Forward	5’- GGGGTTATTGTGAAAGGGTTTATAT-3’
	Reverse	5’- CCAATTCACTTTAATTTTCCAACTC-3’
*ACTB*	Forward	5’-TCACCCACACTGTGCC CATCTACGA-3’
	Reverse	5’-CAGCGGAACCGCTCATTGCCAATGG-3’

*COL1A1*, collagen type I alpha 1; COL1A2, collagen type I alpha 2; *COL2A1*, Collagen Type II Alpha 1 Chain; *COL4A1*, Collagen Type IV Alpha 1 Chain; *COL4A4*, Collagen Type IV Alpha 4 Chain; *COL4A6*, Collagen Type IV Alpha 6 Chain; *COL6A2*, Collagen Type VI Alpha 2 Chain; *PIK3CA*, Phosphatidylinositol-4,5-Bisphosphate 3-Kinase Catalytic Subunit Alpha; *PIK3C*B, Phosphatidylinositol-4,5-Bisphosphate 3-Kinase Catalytic Subunit Beta; *PIK3CD*, Phosphatidylinositol-4,5-Bisphosphate 3-Kinase Catalytic Subunit Delta; *PIK3CG*, Phosphatidylinositol-4,5-Bisphosphate 3-Kinase Catalytic Subunit Gamma; *PIK3R1*, Phosphoinositide-3-Kinase Regulatory Subunit 1; *PIK3R4*, Phosphoinositide-3-Kinase Regulatory Subunit 4; *MAPK1*, Mitogen-Activated Protein Kinase 1; *MAPK3*, Mitogen-Activated Protein Kinase 3; *MAP2K2*, Mitogen-Activated Protein Kinase Kinase 2; *mTOR*, Mechanistic Target of Rapamycin; *ACTB*, β-actin.

### 2.7 Enzyme-linked immunosorbent assay

The protein expression profile was evaluated using ELISA (Abbexa, Cambridge, UK) with the following kits: COL1A1 kit (MyBioSource, Inc., San Diego, CA, USA; cat. no. MBS703198), COL4A1 (MyBioSource, Inc., San Diego, CA, USA; cat. no. MBS8801839), MAPK3 (MyBioSource, Inc., San Diego, CA, USA; cat. no. MBS2515441), PIK3CG (MyBioSource, Inc., San Diego, CA, USA; cat. no. MBS2507349), PIK3R (MyBioSource, Inc., San Diego, CA, USA; cat. no. MBS720875), PIK3CA (MyBioSource, Inc., San Diego, CA, USA; cat. no. MBS3803548), PIK3CD (MyBioSource, Inc., San Diego, CA, USA; cat. no. MBS2020127), and mTOR (MyBioSource, Inc., San Diego, CA, USA; cat. no. MBS2505637).

### 2.8 Statistical analysis

Data analysis was carried out using Statistica 13.0 PL and the Transcriptome Analysis Console (Affymetrix). The Shapiro–Wilk test assessed data normality, with significance set at p < 0.05. One-way ANOVA with Benjamin–Hochberg correction and Tukey’s *post hoc* test, or Student’s t-test, were applied for mean comparisons. Overall survival was plotted using Kaplan–Meier (http://kmplot.com/) (37, 38). We used the log-rank test to evaluate the statistical significance of differences in survival curves between high and low expression groups for specific mRNAs. Additionally, hazard ratios (HRs) with 95% confidence intervals were calculated to quantify the impact of gene expression levels on overall survival (OS). Gene interactions were evaluated using STRING Database 11.0 (accessed May 5, 2024), with enrichment quantified by Log_10_ (observed/expected) and significance evaluated using the false discovery rate, adjusted with the Benjamini–Hochberg method. Gene clustering was performed using the Markov Cluster (MCL) algorithm, with the inflation parameter optimized to identify biologically relevant clusters while maintaining pathway-level resolution (39). Sample size was calculated using a sampling calculator, assuming a 95% confidence interval. Based on approximately 19,620 women diagnosed with breast cancer in Poland in 2019, the recommended sample size was 377. Subtype distribution in this study compared to literature data is as follows: luminal A (32.10% *vs*. 23.7%), luminal B HER2- (38.8% in both), luminal B HER2+ (23.7% *vs*. 14%), HER2+ (8.89% *vs*. 11.2%), and TNBC (10.62% *vs*. 12.3%) (40, 41).

## 3 Results

### 3.1 Microarray profile of PI3K/AKT/mTOR pathway-related genes breast cancer samples in comparison with control tissue

**Figure 1:**
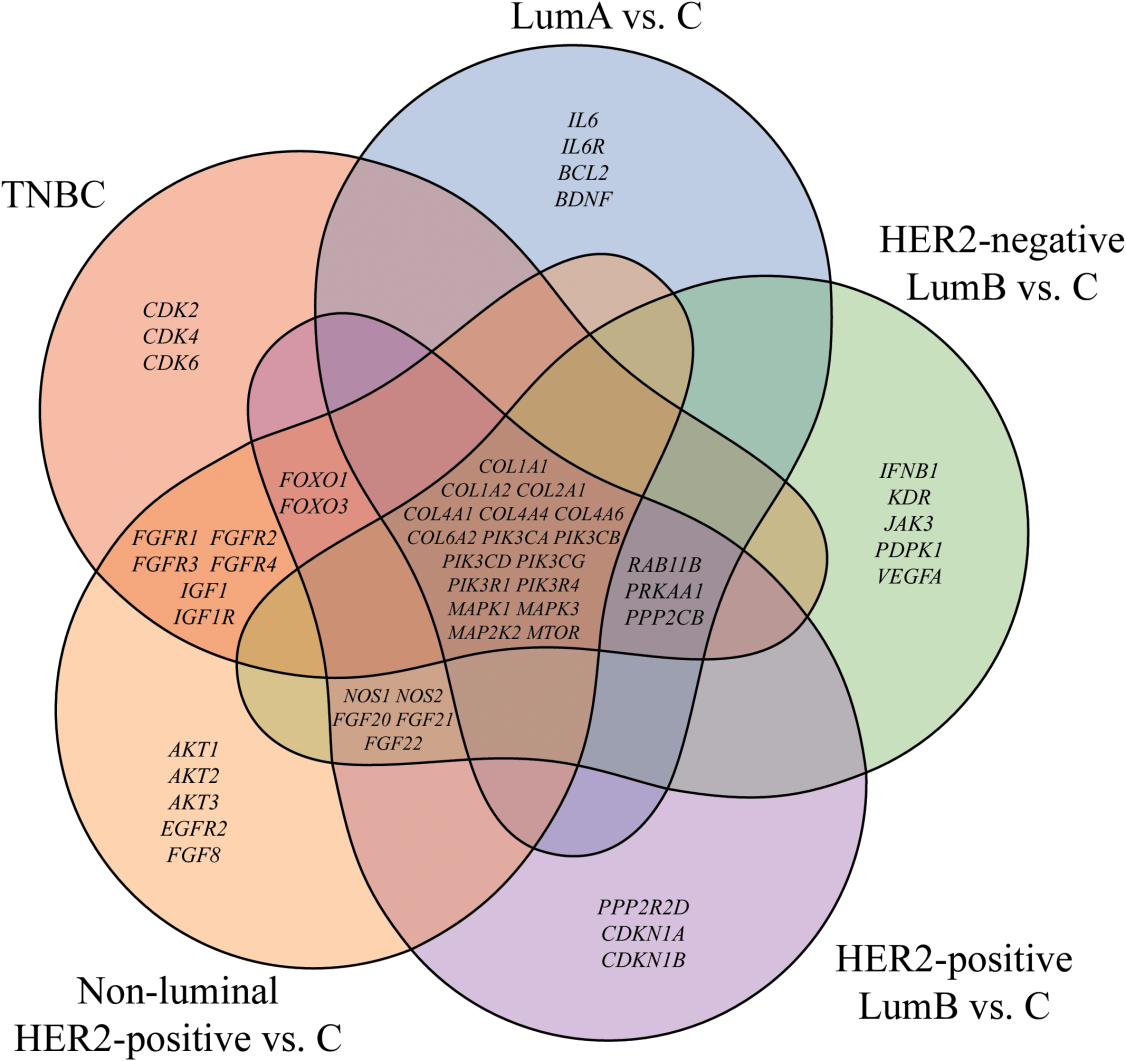
Venn diagram of genes differentiating breast cancer from the control. LumA, luminal A; LumB, luminal B; HER2, human epidermal growth factor receptor 2; TNBC, triple-negative breast cancer; C, control; *COL1A1*, collagen type I alpha 1; COL1A2, collagen type I alpha 2; *COL2A1*, Collagen Type II Alpha 1 Chain; *COL4A1*, Collagen Type IV Alpha 1 Chain; *COL4A4*, Collagen Type IV Alpha 4 Chain; *COL4A6*, Collagen Type IV Alpha 6 Chain; *COL6A2*, Collagen Type VI Alpha 2 Chain; *PIK3CA*, Phosphatidylinositol-4,5-Bisphosphate 3-Kinase Catalytic Subunit Alpha; *PIK3C*B, Phosphatidylinositol-4,5-Bisphosphate 3-Kinase Catalytic Subunit Beta; *PIK3CD*, Phosphatidylinositol-4,5-Bisphosphate 3-Kinase Catalytic Subunit Delta; *PIK3CG*, Phosphatidylinositol-4,5-Bisphosphate 3-Kinase Catalytic Subunit Gamma; *PIK3R1*, Phosphoinositide-3-Kinase Regulatory Subunit 1; *PIK3R4*, Phosphoinositide-3-Kinase Regulatory Subunit 4; *MAPK1*, Mitogen-Activated Protein Kinase 1; *MAPK3*, Mitogen-Activated Protein Kinase 3; *MAP2K2*, Mitogen-Activated Protein Kinase Kinase 2; *mTOR*, Mechanistic Target of Rapamycin.

Out of the 390 mRNAs associated with the PI3K/AKT/mTOR signaling pathway, one-way ANOVA analysis revealed that 55 mRNAs were significantly altered in the study samples compared to control samples (-3.0 < FC > 3.0; p < 0.05). A subsequent Tukey’s *post hoc* analysis identified specific mRNAs distinguishing between breast cancer subtypes, as well as those shared across multiple subtypes. **Figure 1** presents a Venn diagram illustrating the genes characteristic of individual breast cancer subtypes and those overlapping between groups. This visualization underscores the complexity of transcriptional changes, highlighting both subtype-specific and shared molecular features, which are crucial for understanding the diverse biological mechanisms underlying breast cancer. Insights gained from the overlaps include evidence of common pathways contributing to tumor progression across subtypes, while subtype-specific differences reveal potential targets for tailored therapeutic strategies.

**Table 3** further elaborates on these findings, detailing transcriptional activity changes for the 17 mRNAs identified across the five breast cancer subtypes. Notably, significant upregulation of collagen genes, such as *COL1A1*, *COL1A2*, *COL2A1*, *COL4A1*, and *COL4A4*, was observed, particularly in the TNBC and Non-luminal HER2+ subtypes, with fold changes reaching up to 8.12 for *COL2A1* in TNBC. Similarly, genes in the PIK3 pathway, including *PIK3CA*, *PIK3CB*, and *PIK3CD*, exhibited robust upregulation with fold changes for *PIK3CA* and *PIK3CB* peaking at 11.98 and 12.76, respectively, in TNBC. Additionally, MAPK and mTOR pathway genes showed marked elevation, exemplified by *MAP2K2*’s fold change of 14.88 in TNBC and mTOR’s consistent upregulation across all subtypes, reaching 9.10 in TNBC. Collectively, these findings suggest that the PI3K/AKT/mTOR, MAPK, and collagen-related pathways play a particularly prominent role in the aggressive behavior of TNBC and Non-luminal HER2+ breast cancer subtypes.

**Table 3:** Variances in the expression profile of genes differentiating tumor samples compared to control tissues (-3.0<FC>3.0; p <0.05).

ID	mRNA	Luminal A *vs*. control	Luminal B HER2- *vs*. control	Luminal B ERr2+ *vs*. control	Non-luminal HER2+ *vs*. control	TNBC *vs*. control
202311_s_at	*COL1A1*	3.41	3.84	5.11	6.9	7.51
217430_x_at		3.77	4.12	5.38	5.68	6.56
202310_s_at		2.98	3.55	4.89	5.17	6.11
202312_s_at		3.09	3.45	4.78	5.67	6.18
202404_s_at	*COL1A2*	3.83	4.06	5.09	5.63	7.74
202403_s_at		3.29	4.13	5.12	5.71	7.89
217404_s_at	*COL2A1*	3.12	3.76	5.12	5.98	8.12
213492_at		3.45	3.98	4.98	5.99	8.18
211981_at	*COL4A1*	2.98	3.43	5.12	6.13	7.87
211980_at		3.34	3.45	5.17	6.18	7.81
214602_at	*COL4A4*	3.56	3.98	4.91	6.81	8.11
213992_at	*COL4A6*	2.81	3.09	4.19	5.14	5.19
211473_s_at		2.90	3.12	4.55	5.22	5.29
210945_at		2.81	3.11	4.51	5.19	5.11
213290_at	*COL6A2*	3.56	3.89	4.98	5.13	6.81
209156_s_at		3.45	3.91	5.01	5.16	6.71
204369_at	*PIK3CA*	4.56	6.17	8.18	10.12	11.98
202838_s_at	*PIK3CB*	5.12	7.16	9.19	10.56	12.76
212688_at	*PIK3CD*	3.41	4.78	7.81	8.11	10.19
217620_s_at		3.12	4.56	7.12	8.71	10.91
206370_at	*PIK3CG*	2.81	4.67	7.87	9.18	10.22
206369_s_at		3.01	4.56	7.88	9.19	10.12
212240_s_at	*PIK3R1*	-3.45	-4.58	-5.99	-7.18	-8.65
212239_at		-3.41	-4.56	-6.02	-7.14	-8.98
212249_at		-3.45	-4.61	-5.41	-7.41	-8.78
216436_at	*PIK3R4*	2.18	2.76	3.45	4.56	6.09
216752_at		2.23	2.98	3.65	4.71	5.99
212740_at		2.71	3.01	3.61	4.67	6.12
212271_at	*MAPK1*	4.12	5.98	7.12	8.19	9.10
208351_s_at		4.19	5.78	7.32	8.11	9.12
212046_x_at	*MAPK3*	4.13	5.67	7.18	9.18	12.11
213490_s_at	*MAP2K2*	9.87	11.09	12.19	13.45	14.88
202424_at		10.01	11.87	12.11	13.16	14.65
213487_at		9.98	11.52	12.18	13.27	14.76
202288_at	*MTOR*	4.12	4.78	7.16	8.12	9.10
215381_at		4.15	4.76	7.18	8.17	9.12

ID, number of the probe; LumA, luminal A; LumB, luminal B; HER2, human epidermal growth factor receptor 2; TNBC, triple-negative breast cancer; C, control; COL1A1, collagen type I alpha 1; COL1A2, collagen type I alpha 2; COL2A1, Collagen Type II Alpha 1 Chain; COL4A1, Collagen Type IV Alpha 1 Chain; COL4A4, Collagen Type IV Alpha 4 Chain; COL4A6, Collagen Type IV Alpha 6 Chain; COL6A2, Collagen Type VI Alpha 2 Chain; PIK3CA, Phosphatidylinositol-4,5-Bisphosphate 3-Kinase Catalytic Subunit Alpha; PIK3CB, Phosphatidylinositol-4,5-Bisphosphate 3-Kinase Catalytic Subunit Beta; PIK3CD, Phosphatidylinositol-4,5-Bisphosphate 3-Kinase Catalytic Subunit Delta; PIK3CG, Phosphatidylinositol-4,5-Bisphosphate 3-Kinase Catalytic Subunit Gamma; PIK3R1, Phosphoinositide-3-Kinase Regulatory Subunit 1; PIK3R4, Phosphoinositide-3-Kinase Regulatory Subunit 4; MAPK1, Mitogen-Activated Protein Kinase 1; MAPK3, Mitogen-Activated Protein Kinase 3; MAP2K2, Mitogen-Activated Protein Kinase Kinase 2; MTOR, Mechanistic Target of Rapamycin.

### 3.2 Expression pattern of PI3K/AKT/mTOR pathway-related genes in breast cancer samples compared to control tissue analyzed by qRT-PCR

To validate the microarray findings, we conducted qRT-PCR analysis of 17 mRNAs across five distinct breast cancer subtypes: Luminal A, Luminal B HER2-, Luminal B ER2+, Non-luminal HER2+, and TNBC. The qRT-PCR results demonstrated a high level of concordance with the microarray data, underscoring the technical reliability and robustness of our initial observations. his technical concordance was confirmed by consistent fold-change patterns across both methods, validating the reliability of the identified molecular markers. Consistent with microarray findings, qRT-PCR revealed significant upregulation of collagen genes across all subtypes, with TNBC and Non-luminal HER2+ subtypes exhibiting the most pronounced increases. In particular, *COL1A1* and *COL6A2* showed notable fold changes, reaching statistical significance in TNBC, which supports the hypothesis that collagen-related pathways may play an enhanced role in the aggressiveness and structural remodeling associated with these more invasive subtypes.

The analysis further showed that genes associated with the PIK3 pathway were consistently and significantly upregulated across several subtypes. In Non-luminal HER2+ and TNBC, *PIK3CA* and *PIK3CD* displayed the highest fold changes, with values reaching statistical thresholds indicative of a substantial impact on PI3K/AKT signaling. These findings reinforce the importance of the PIK3 pathway in promoting tumor growth and survival in these aggressive subtypes and highlight its potential as a therapeutic target.

In addition to collagen and PIK3 pathway genes, qRT-PCR results also confirmed the upregulation of MAPK and mTOR signaling pathway genes across all breast cancer subtypes. Among these, *mTOR* and *MAP2K2* showed particularly high and statistically significant fold changes in TNBC and Non-luminal HER2+ samples, suggesting a crucial role for these pathways in driving the proliferation and metabolic activity characteristic of these subtypes. Specifically, the upregulation of *MAP2K2* in TNBC, with fold changes substantially higher than in other subtypes, suggests a subtype-specific enhancement of MAPK signaling that could contribute to the observed aggressiveness of TNBC.

Consistent with microarray findings, qRT-PCR revealed significant upregulation of collagen genes across all subtypes, with TNBC and Non-luminal HER2+ subtypes exhibiting the most pronounced increases. *COL1A1* showed particularly high fold changes in these subtypes, reinforcing its potential role in tumor aggressiveness and extracellular matrix remodeling.

This strong concordance between microarray and qRT-PCR data for COL1A1 validates its reliability as a molecular marker, supporting its role in promoting tumor invasiveness in these more aggressive subtypes.

Similarly, *MAPK*3 displayed significant and consistent upregulation in TNBC and Non-luminal HER2+ subtypes across both qRT-PCR and microarray analyses. This correlation highlights MAPK3’s involvement in the MAPK signaling pathway, known for regulating cell proliferation and survival, especially in aggressive cancer phenotypes. The robust expression correlation of MAPK3 between the two methods confirms its importance as a molecular marker, underscoring the role of MAPK signaling in the aggressiveness of TNBC and HER2+ subtypes.

**Figure 2:**
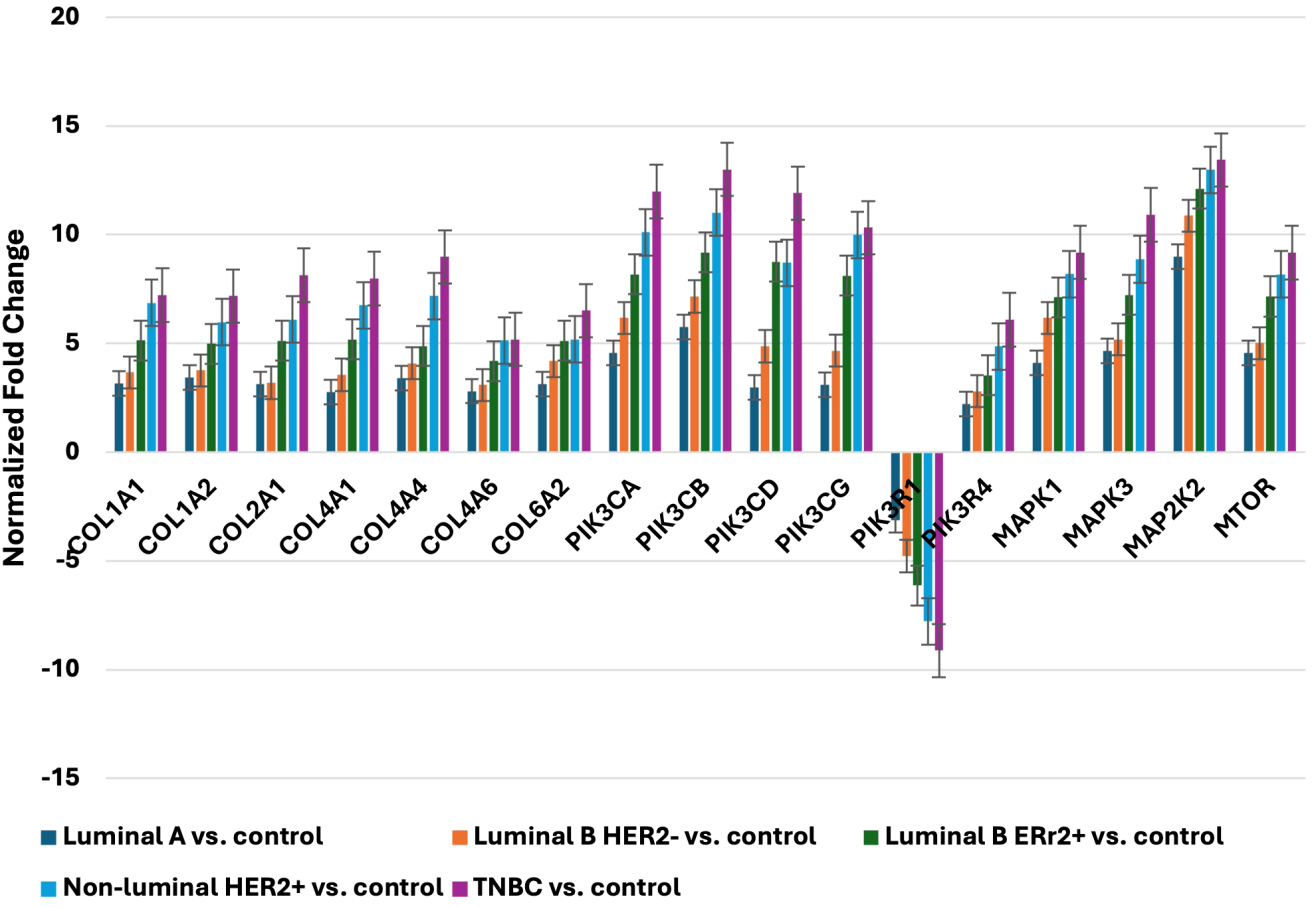
Expression profile of selected genes determined by qRT-PCR. LumA, luminal A; LumB, luminal B; HER2, human epidermal growth factor receptor 2; TNBC, triple-negative breast cancer; C, control; *COL1A1*, collagen type I alpha 1; *COL1A2*, collagen type I alpha 2; *COL2A1*, Collagen Type II Alpha 1 Chain; *COL4A1*, Collagen Type IV Alpha 1 Chain; *COL4A4*, Collagen Type IV Alpha 4 Chain; *COL4A6*, Collagen Type IV Alpha 6 Chain; *COL6A2*, Collagen Type VI Alpha 2 Chain; *PIK3CA*, Phosphatidylinositol-4,5-Bisphosphate 3-Kinase Catalytic Subunit Alpha; *PIK3CB*, Phosphatidylinositol-4,5-Bisphosphate 3-Kinase Catalytic Subunit Beta; *PIK3CD*, Phosphatidylinositol-4,5-Bisphosphate 3-Kinase Catalytic Subunit Delta; *PIK3CG*, Phosphatidylinositol-4,5-Bisphosphate 3-Kinase Catalytic Subunit Gamma; *PIK3R1*, Phosphoinositide-3-Kinase Regulatory Subunit 1; *PIK3R4*, Phosphoinositide-3-Kinase Regulatory Subunit 4; *MAPK1*, Mitogen-Activated Protein Kinase 1; *MAPK3*, Mitogen-Activated Protein Kinase 3; *MAP2K2*, Mitogen-Activated Protein Kinase Kinase 2; *mTOR*, Mechanistic Target of Rapamycin.

The consistency observed between microarray and qRT-PCR data across these genes confirms the validity of our findings and highlights the technical rigor of the study. These results strongly indicate that the PI3K/AKT, MAPK, and mTOR pathways are disproportionately active in TNBC and Non-luminal HER2+ subtypes, likely contributing to the aggressive clinical phenotypes observed in these groups. Overall, the qRT-PCR data corroborates the microarray findings, particularly in the context of subtype-specific upregulation patterns, which not only underscores the distinct molecular characteristics of each breast cancer subtype but also points to potential therapeutic avenues targeting these elevated pathways (**Figure 2**).

### 3.3 Prediction of PI3K/AKT/mTOR pathway genes expression regulation by miRNA

Notably, miR-129 is predicted to downregulate *COL1A1*, with the most substantial suppression observed in the TNBC subtype (−3.31 ± 0.15*). PIK3R1* is shown to be markedly downregulated by miR-190a-3p, with the greatest reduction in TNBC (−4.01 ± 0.31). Additionally, miR-4729 is associated with consistent downregulation of *mTOR* across all subtypes, with TNBC exhibiting the largest fold change (−3.45 ± 0.19). Similarly, *PIK3CA* is significantly downregulated by miR-19a-3p, with the most pronounced suppression in TNBC (−5.14 ± 0.18). In the case of *PIK3CD*, miR-30d-5p is predicted to induce downregulation, with the highest fold change also observed in TNBC (−3.98). The results of selected miRNAs expression was presented in **Table 4**.

**Table 4:** Expression of miRNAs potentially involved in the regulation of selected genes (p<0.05; FC > 2 or < -2).

mRNA	miRNA	Target score	Fold change				
			LumA *vs*. C	HER2-negative LumB *vs*. C	HER2-positive LumB *vs*. C	HER2-positive *vs*. C	TNBC *vs*. C
***COL1A1***	miR-129	90	−2.04 ± 0.21	−2.22 ± 0.43	−2.25 ± 0.18	−2.98 ± 0.14	−3.31 ± 0.15
***COL4A1*** ***MAPK3*** ***PIK3CG*** ***PIK3R1***	miR-190a-3p	100 89 100 100	-2.56 ± 0.32	-3.13 ± 0.19	-3.45 ± 0.12	-3.61 ± 0.67	-4.01 ± 0.31
***mTOR***	miR-4729	98	−2.19 ± 0.19	−2.91 ± 0.76	−3.19 ± 0.32	−3.21 ± 0.71	-3.45 ± 0.19
***PIK3CA***	mir-19a-3p	98	-3.18 ± 0.43	−3.87 ± 0.61	−4.66 ± 0.19	−4.98 ± 0.12	-5.14 ± 0.18
***PIK3CD***	miR-30d-5p	93	−2.99	−3.45	−3.66	−3.91	−3.98

LumA, luminal A; LumB, luminal B; HER2, human epidermal growth factor receptor 2; TNBC, triple-negative breast cancer; COL1A1, Collagen Type I Alpha 1 Chain; COL4A1, Collagen Type IV Alpha 1 Chain; MAPK3, Mitogen-Activated Protein Kinase 3; PIK3CG, Phosphatidylinositol-4,5-Bisphosphate 3-Kinase Catalytic Subunit Gamma; PIK3R1, Phosphoinositide-3-Kinase Regulatory Subunit 1; MTOR, Mechanistic Target of Rapamycin; PIK3CA, Phosphatidylinositol-4,5-Bisphosphate 3-Kinase Catalytic Subunit Alpha; PIK3CD, Phosphatidylinositol-4,5-Bisphosphate 3-Kinase Catalytic Subunit Delta.

### 3.4 Concentration of selected proteins in breast cancer tissues and control at the protein level

In the next step, we analyzed variances in the concentration of protein encoded by mRNA potentially regulated by miRNA. The analysis of protein concentrations encoded by mRNA potentially regulated by miRNA revealed significant variances across breast cancer subtypes. All proteins measured, including COL1A1, COL4A1, MAPK3, PIK3CG, PIK3CA, PIKCD, PIK3R1, and mTOR, exhibited progressive increases in concentration from the control group through the LumA, HER2-negative LumB, HER2-positive LumB, HER2-positive, and TNBC subtypes, with the highest levels observed in TNBC. Notably, COL1A1 and COL4A1 concentrations in TNBC reached 24.33 ng/mL and 25.12 ng/mL, respectively, compared to 3.7 ng/mL and 5.14 ng/mL in the control. Similarly, PIK3CG and mTOR levels increased sharply, suggesting a potential role of miRNA regulation in driving protein expression, which intensifies with cancer aggressiveness. Each increase across subtypes was statistically significant (*p < 0.05), underscoring miRNA’s possible involvement in the regulation of protein expression in different breast cancer phenotypes (**Table 5**; p < 0.05).

**Table 5:** Concentration of selected proteins in breast cancer subtypes and control group (p<0.05).

Protein [ng/mL]	Control	LumA	HER2-negative LumB	HER2-positive LumB	HER2-positive	TNBC
**COL1A1**	3.7 ± 0.31	7.91 ± 0.19*	11.98 ± 0.27*	14.42 ± 0.27*	19.38 ± 0.34*	24.33 ± 0.31*
**COL4A1**	5.14 ± 0.11	12.34 ± 0.29*	16.78 ± 0.41*	18.98 ± 0.31*	20.91 ± 0.19*	25.12 ± 0.41*
**MAPK3**	3.12 ± 0.12	6.18 ± 0.19*	9.18 ± 0.91*	10.01 ± 0.12*	12.19 ± 0.71*	16.18 ± 0.42*
**PIK3CG**	0.23± 0.08	1.09 ± 0.1I*	1.87 ± 0.21*	3.45 ± 0.12*	4.56 ± 0.21*	7.19 ± 0.11*
**PIK3CA**	2.01 ± 0.11	6.18 ± 0.62*	9.18 ± 0.56*	10.11 ± 0.21*	11.76 ± 0.45*	14.56 ± 0.82*
**PIKCD**	0.41 ± 0.03	2.35 ± 0.34*	4.16 ± 0.16*	5.77 ± 0.18*	9.18 ± 0.28*	10.11 ± 0.47*
**PIK3R1**	0.54 ± 0.02	1.98 ± 0.12*	2.98 ± 0.51*	5.67 ± 0.19*	6.17 ± 0.19*	7.04 ± 0.16*
**mTOR**	0.91 ± 0.07	2.87 ± 0.19*	3.19 ± 0.16*	4.56 ± 0.19*	4.94 ± 0.11*	5.76 ± 0.17*

LumA, luminal A; LumB, luminal B; HER2, human epidermal growth factor receptor 2; TNBC, triple-negative breast cancer; C, control; COL1A1, Collagen Type I Alpha 1 Chain; COL4A1, Collagen Type IV Alpha 1 Chain; MAPK3, Mitogen-Activated Protein Kinase 3; PIK3CG, Phosphatidylinositol-4,5-Bisphosphate 3-Kinase Catalytic Subunit Gamma; PIK3R1, Phosphoinositide-3-Kinase Regulatory Subunit 1; MTOR, Mechanistic Target of Rapamycin; PIK3CA, Phosphatidylinositol-4,5-Bisphosphate 3-Kinase Catalytic Subunit Alpha; PIK3CD, Phosphatidylinositol-4,5-Bisphosphate 3-Kinase Catalytic Subunit Delta. *p < 0.05 *vs*. control.

### 3.5 Results of the STRING database analysis

**Figure 3:**
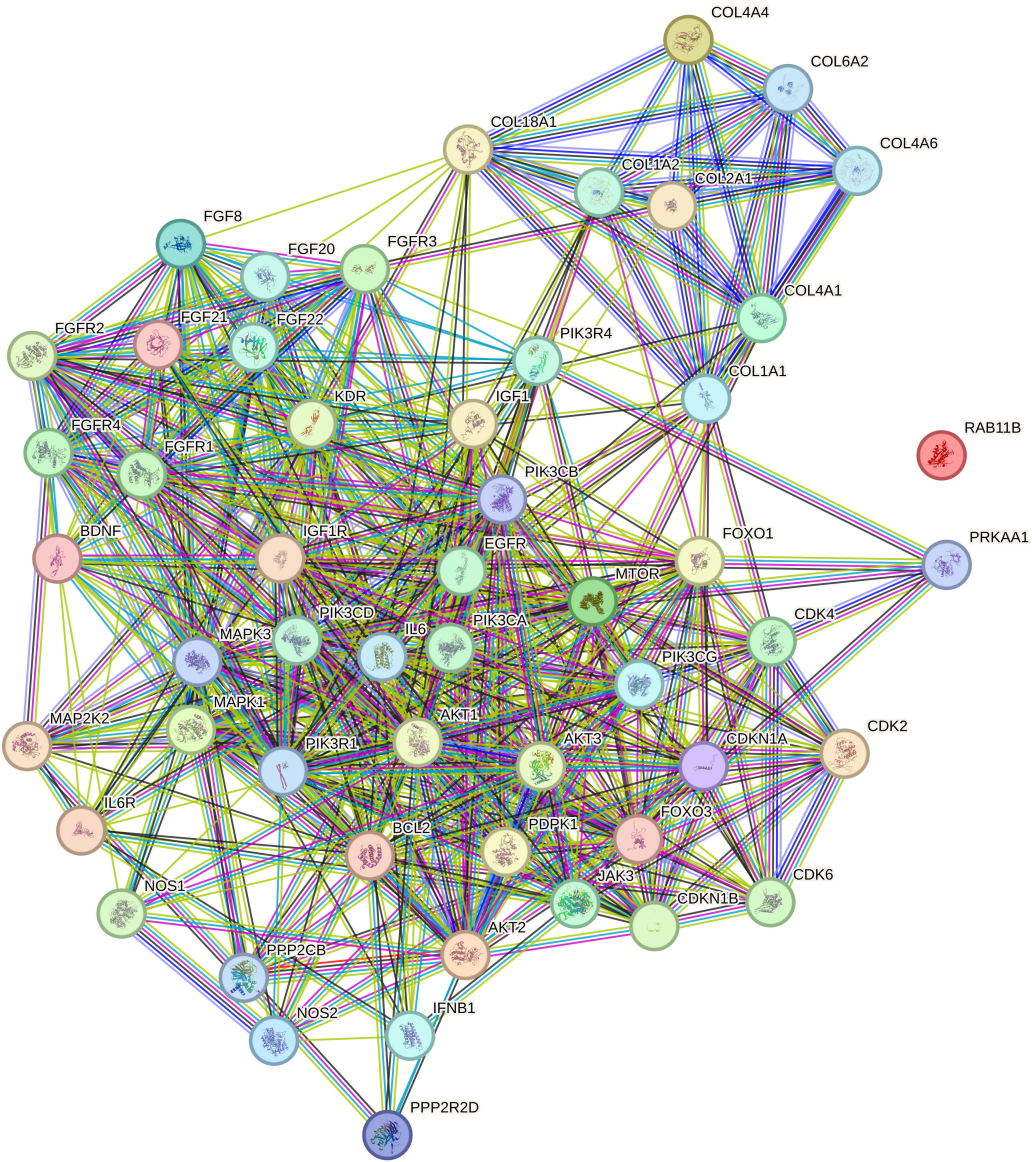
Relationship network for the selected PI3K/AKT/mTOR pathway differentiation genes generated in the STRING database. COL1A1, collagen type I alpha 1; COL1A2, collagen type I alpha 2; COL2A1, Collagen Type II Alpha 1 Chain; COL4A1, Collagen Type IV Alpha 1 Chain; COL4A4, Collagen Type IV Alpha 4 Chain; COL4A6, Collagen Type IV Alpha 6 Chain; COL6A2, Collagen Type VI Alpha 2 Chain; PIK3CA, Phosphatidylinositol-4,5-Bisphosphate 3-Kinase Catalytic Subunit Alpha; PIK3CB, Phosphatidylinositol-4,5-Bisphosphate 3-Kinase Catalytic Subunit Beta; PIK3CD, Phosphatidylinositol-4,5-Bisphosphate 3-Kinase Catalytic Subunit Delta; PIK3CG, Phosphatidylinositol-4,5-Bisphosphate 3-Kinase Catalytic Subunit Gamma; PIK3R1, Phosphoinositide-3-Kinase Regulatory Subunit 1; PIK3R4, Phosphoinositide-3-Kinase Regulatory Subunit 4; MAPK1, Mitogen-Activated Protein Kinase 1; MAPK3, Mitogen-Activated Protein Kinase 3; MAP2K2, Mitogen-Activated Protein Kinase Kinase 2; mTOR, Mechanistic Target of Rapamycin.

The STRING database network analysis visualizes the interactions among 55 nodes, representing genes or proteins, and 560 edges, indicating known or predicted interactions between them (**Figure 3**). This highly connected network is characterized by an average node degree of 20.4, meaning each node interacts with approximately 20 other nodes on average. The high average local clustering coefficient of 0.719 suggests a strong tendency for interconnected nodes to form clusters, indicating that these proteins are likely involved in common biological pathways or processes.

Notably, the expected number of edges in a random network with the same number of nodes would be only 151, whereas the observed number of edges is 560, which is significantly greater than expected by chance. This is further supported by the PPI (Protein-Protein Interaction) enrichment p-value of <1.0e-16, suggesting that the observed interactions are statistically significant and that the proteins likely share functional relationships.

Within this dense network, several key clusters emerge, characterized by high interaction scores that signify strong functional associations among the proteins. The most connected nodes, such as PIK3CA and mTOR, serve as central hubs within the network. PIK3CA, a catalytic subunit of the PI3K enzyme, exhibits numerous interactions with other signaling molecules, reflecting its pivotal role in the PI3K/AKT/mTOR pathway. Similarly, mTOR is a crucial kinase involved in cell growth, proliferation, and survival, and its high connectivity underscores its integral position in multiple signaling cascades.

The prominence of these nodes suggests potential pathway crosstalk between the PI3K/AKT/mTOR pathway and other critical signaling pathways, such as the MAPK pathway. For instance, interactions between PIK3CA, mTOR, and MAP2K2 (also known as MEK2) indicate a convergence of signaling networks that regulate key cellular processes. This crosstalk may contribute to the complexity of cancer progression and highlights the potential for combined therapeutic strategies targeting multiple pathways.

Additionally, clusters involving collagen genes like COL1A1 and COL1A2 demonstrate high connectivity, suggesting their collective involvement in extracellular matrix organization and interactions with cell signaling pathways. These interactions may influence tumor microenvironment dynamics, affecting cancer cell invasion and metastasis.

Overall, the network illustrates a dense web of interactions with significant hubs and clusters that are essential for understanding the molecular underpinnings of breast cancer subtypes. The high interaction scores and connectivity of nodes like PIK3CA and mTOR emphasize their roles in oncogenic signaling and potential as targets for therapeutic intervention. These insights reflect robust connectivity in the dataset and point to the significant roles of these proteins in the broader biological processes under investigation, such as cancer development and progression. The identification of key clusters and pathway crosstalk provides a deeper understanding of the complex signaling networks at play, offering avenues for future research and potential strategies for targeted treatment.

### 3.6 Overall survive analysis

**Figure 4:**
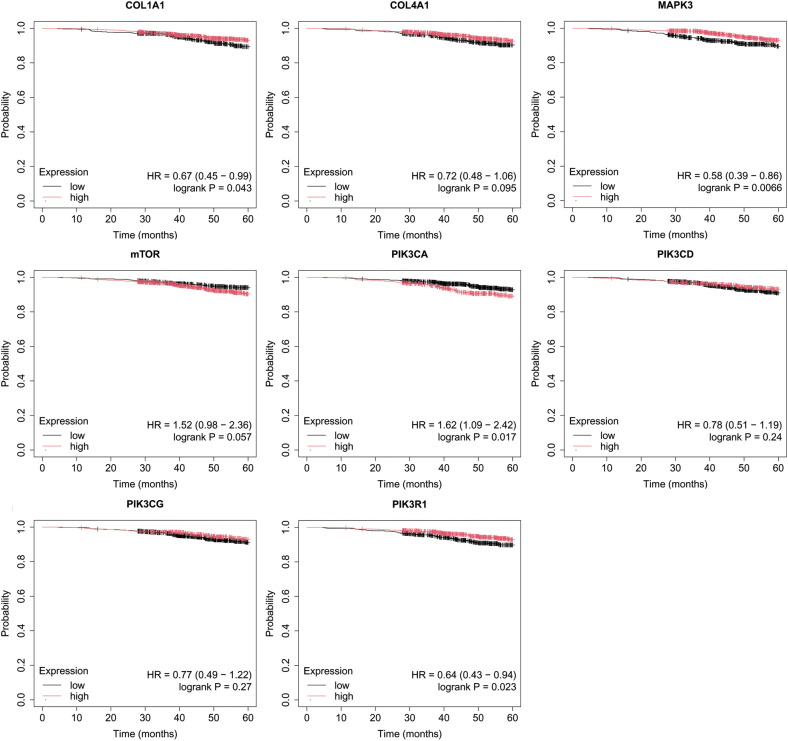
Overall survival analysis for luminal A subtype.

**Figure 5:**
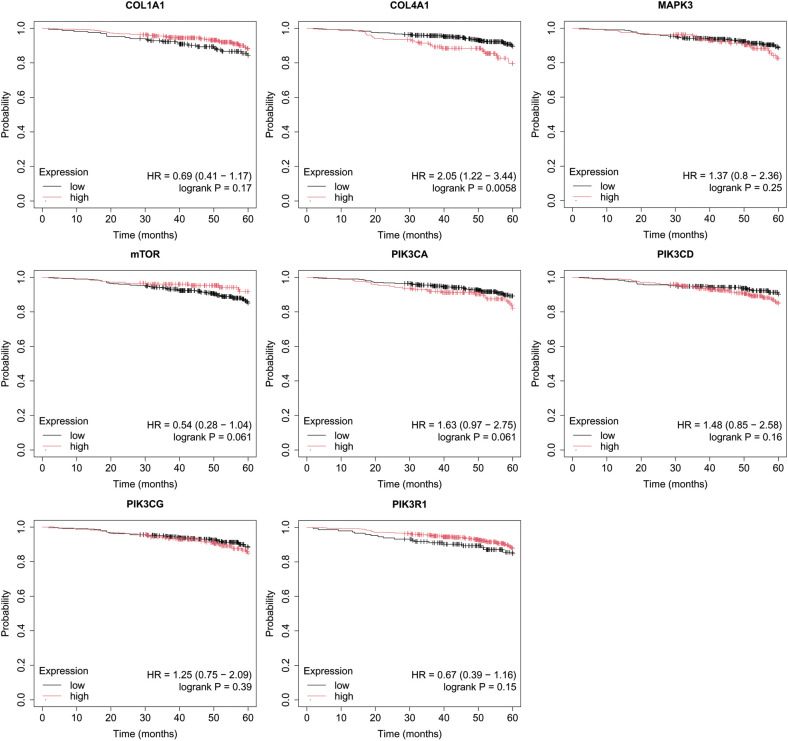
Overall survival analysis for luminal B HER2− subtype.

**Figure 6:**
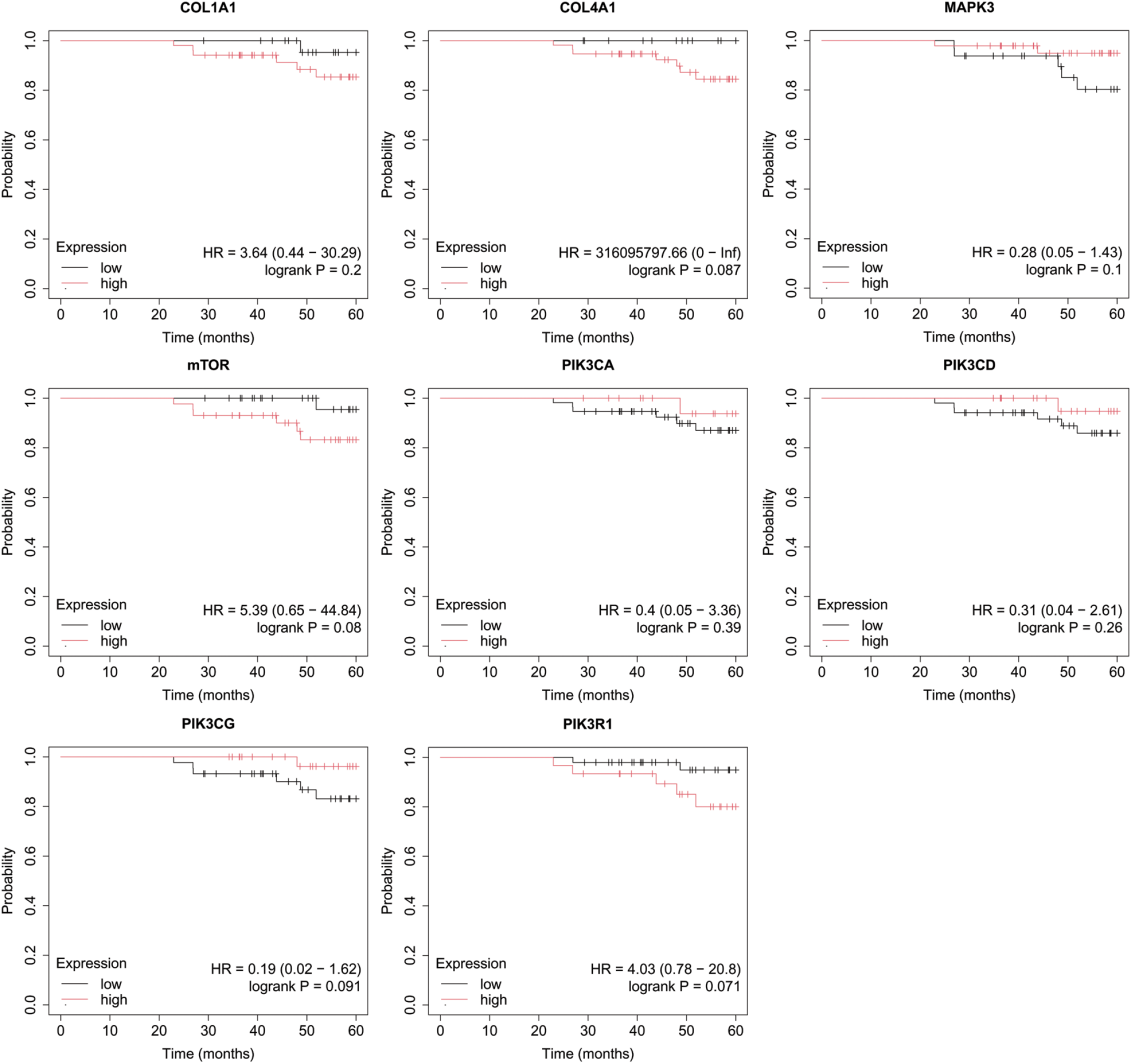
Overall survival analysis for luminal B HER2+ subtype.

**Figure 7:**
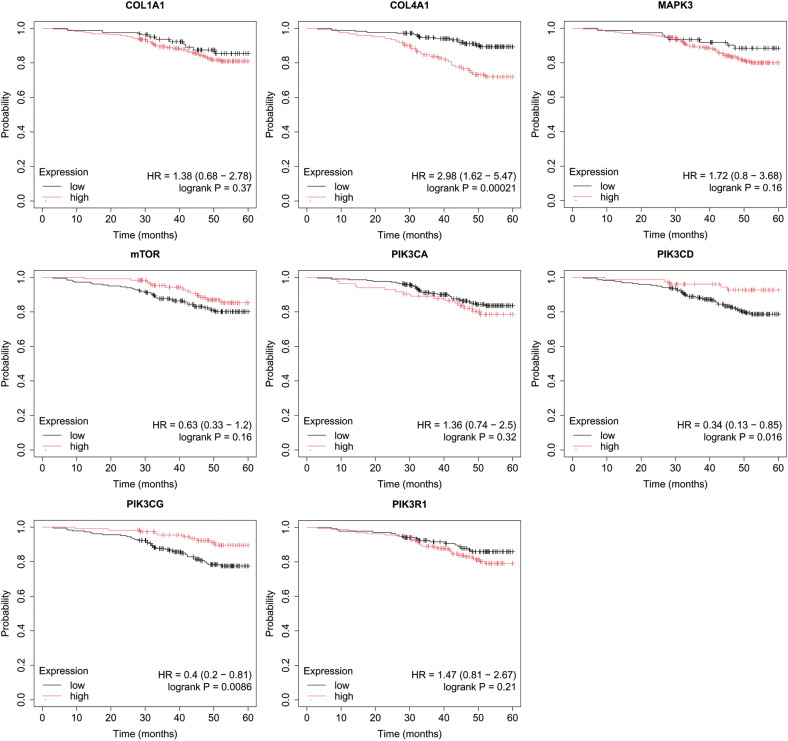
Overall survival analysis for non-luminal HER2+ cancers subtype.

**Figure 8:**
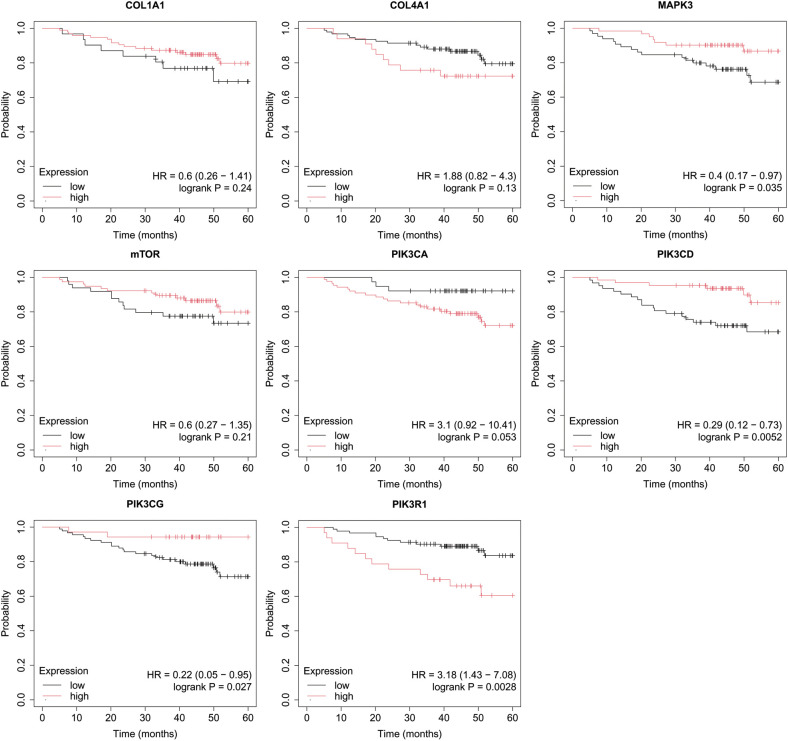
Overall survival analysis for TNBC subtype.

Overall survival (OS) analysis was conducted for the studied mRNAs using the Kaplan–Meier plotter, with the results presented in **Figures 4**–**8**. This analysis provides insight into the association between the expression levels of specific mRNAs and patient survival outcomes. The Kaplan–Meier curves allow for the comparison of survival times between different expression levels of these mRNAs, helping to identify which genes may serve as potential prognostic markers in breast cancer.

In the case of the luminal A subtype, high PIK3CA expression, together with low COL1A1, MAPK3 and PIK3R1 activity, promoted worse OS in patients with luminal A (**Figure 4**).

In turn, in luminal B HER2-, only high COL4A1 activity was important for OS (**Figure 5**).

In luminal B HER2+ samples, the expression of the analyzed genes was not important for OS (**Figure 6**).

In the case of the non-luminal HER2+ subtype, high COL4A1, together with low PIK3CD, and PIK3CG activity, promoted worse OS in patients (**Figure 7**).

In turn, high COL4A1, PIK3R1 expression and low MAPK3, PIK3CD, PIK3CG activity were important for OS in patients with TNBC (**Figure 8**).

## 4 Discussion

Omics techniques, including microarray technology, provide an opportunity to explore signaling pathways and the associated genes encoding proteins relevant to a given disease, as well as to assess treatment effectiveness. This approach enables the identification of complementary molecular markers that allow for early diagnosis of a disease, even before phenotypic manifestations appear, and aids in selecting personalized therapies. Moreover, integrating microarray analysis with bioinformatic approaches facilitates the identification of genes related to specific signaling pathways or biological processes that may not have been previously linked to the condition (42–44).

In our analysis, we adopted a strategy to identify mRNAs related to the PI3K/AKT/mTOR pathway, which differentiate between the five subtypes of breast cancer, as well as transcripts common to all breast cancer types. For the shared genes, we performed a predictive miRNA analysis to identify miRNAs that potentially regulate the expression of these common mRNAs. We also assessed the expression pattern of the proteins encoded by these genes across each breast cancer subtype in comparison to control tissue—non-cancerous tissue obtained during surgery. Based on this analysis, we demonstrated a correlation between the expression of the following mRNAs: *COL1A1, COL4A1, MAPK3, PIK3CG, PIK3CA, PIK3CD, PIK3R1*, and *mTOR* and the regulatory impact of the identified miRNAs on their transcriptional activity. Overexpression of all analyzed mRNA transcripts, expect *PIK3R1* was observed in cancerous tissues compared to controls, with the highest increase in transcriptional activity noted in triple-negative breast cancer (TNBC) compared to other breast cancer types.

In the tumor microenvironment, cancer-associated fibroblasts (CAFs) play a key role in reorganizing and cross-linking COL1A1, leading to its accumulation and the stiffening of the stroma. This, in turn, facilitates cancer cell invasion and migration (45). Liu et al. found that COL1A1 is overexpressed in breast cancer, particularly in ER+ tumors, and is linked to poorer survival outcomes. However, they also observed that patients with elevated COL1A1 levels responded better to cisplatin-based chemotherapy (46). Additionally, Ma et al. demonstrated that down-regulating COL1A1 can suppress tumor growth by inhibiting CAF activation and extracellular matrix (ECM) remodeling in the tumor microenvironment (47). Our miRNA prediction suggests that reduced levels of miR-129 may be linked to the overexpression of COL1A1.

Among the miRNAs analyzed, miR-129 emerged as a critical regulator with dual functionality. Previous studies have shown that miR-129–5p is significantly upregulated in the serum of acute myeloid leukemia (AML) patients compared to normal controls (48). Similarly, diffuse large B-cell lymphoma (DLBCL) patients exhibit elevated levels of miR-129 (49). Interestingly, different mature forms of miR-129 display diverse expressions and functions in human cancers. For instance, miR-129–3p, but not miR-129–5p, is frequently attenuated in clear cell renal cell cancer (ccRCC), suggesting a context-dependent regulatory role (50). Meng et al. reported that miR-129 levels are decreased in breast cancer, and its activity was found to inhibit cancer cell proliferation (51). Similarly, studies by Zeng et al. and Tang et al. observed low miR-129 levels in breast cancer, with its upregulation shown to reduce adriamycin resistance (52, 53). Notably, Li et al. demonstrated that miR-129 inhibits the growth and migration of triple-negative breast cancer (TNBC) cells (54). In contrast, Serijono et al. identified miR-129 as an oncogene in TNBC, where its overexpression targets laminins (55). In this study, miR-129’s predicted regulation of COL1A1 underscores its potential role in extracellular matrix remodeling and tumor invasiveness, particularly in aggressive subtypes like TNBC and Non-luminal HER2+ breast cancer.

Also, in the case of, COL4A1, we noted an overexpression of this transcript in the test sections compared to controls, with the highest found for TNBC. *COL4A1* is a key antiangiogenic gene regulated by p53 in human adenocarcinoma cells. The p53 protein directly promotes the transcription of *COL4A1* by binding to an enhancer region located 26 kilobase pairs downstream of the gene’s 3′ end (56). *COL4A1* encodes a collagen IV molecule, plays a crucial role in mediating cell-to-cell interactions (57). Jin et al. also reported overexpression of COL4A1 in invasive ductal breast carcinoma and demonstrated that assessing its expression has predictive value (58). These findings align with earlier studies indicating that COL4A1-encoded collagen IV can be regulated by P4HA2, playing a role in tumor growth and metastasis (59). Moreover, COL4A1 was found to be one of the most significantly upregulated genes during the development of the avian blood-gas barrier (60). Additionally, Wang et al. indicated that COL4A1 serves as a prognostic biomarker for breast cancer patients who have undergone neoadjuvant chemotherapy (61). Furthermore, our microarray analysis of the profile of genes related to the PI3K/AKT/mTOR pathway and the miRNAs involved in regulating their expression indicated that also the MAPK3 gene plays an important role in the cascade in question in the context of breast cancer. The MAPK (Mitogen-Activated Protein Kinase) and PI3K-AKT-mTOR pathways play key roles in controlling critical cellular functions such as proliferation and survival in breast cancer. These pathways have long been recognized as influential in shaping breast cancer behavior and show significant interactions with the estrogen receptor (ER) pathway (62, 63). This cross-talk is particularly evident in the development of tamoxifen resistance in breast cancer (64). Enhanced EGFR signaling via the MAPK pathway is commonly observed both in clinical settings and in cancer cell lines that have become resistant to endocrine therapies (65). Furthermore, MAPK pathway activation is linked to a higher risk of metastasis (66). Given that signaling networks integrate various upstream signals, targeting MEK has emerged as a promising cancer therapy approach (67). Tumors with RAS/RAF mutations appear more responsive to MEK inhibition, although this response is not consistent across all cases. Additionally, mutations in PIK3CA, which activate the PI3K-AKT-mTOR pathway, are common in breast cancer (68).

Nonetheless, Deng et al. demonstrated that the MAPK1/3 kinase is involved in the proteasomal degradation of ULK1 via ubiquitination, mediated by the E3 ligase BTRC, contributing to the deregulation of ULK1 in breast cancer. The researchers further noted that trametinib, a specific inhibitor of the MAP2K/MEK-MAPK1/3 pathway, which has been FDA-approved for the treatment of metastatic melanoma harboring the BRAF V600E mutation, significantly reduced breast cancer bone metastasis and extended survival in mice with wild-type ULK1 breast cancer xenografts. However, this effect was not observed in mice with ULK1 knockout breast cancer xenografts. Importantly, they also found a negative correlation between MAPK1/3 activation and ULK1 expression in primary breast cancer tissues (69). Given that the RAF-MAP2K/MEK-MAPK1/3 signaling pathway is frequently activated in breast cancer, particularly in TNBC, the common downregulation of ULK1 in breast cancer supports the rationale for using MAP2K/MEK-MAPK1/3 pathway inhibitors to treat breast cancer bone metastasis (70). In preclinical models, trametinib has demonstrated efficacy in reducing breast cancer metastasis and improving survival in mice. For instance, trametinib effectively inhibited the growth of TNBC cells, both sensitive and resistant to other treatments. This inhibition also correlated with increased apoptosis, or programmed cell death, indicating a potential therapeutic strategy for targeting TNBC through MAPK pathway blockade (71, 72). Moreover, clinical and preclinical studies suggest that combining MEK inhibitors with other treatments, such as PI3K inhibitors, could synergistically enhance anti-cancer effects. This co-targeting approach is particularly promising in tumors where both MAPK and PI3K pathways are activated.​ These findings underscore the importance of MAPK3 activation in breast cancer progression and the potential for MAPK pathway inhibitors like trametinib as viable treatment options (72).

The last part of selected genes are strictly related PI3K/AKT/mTOR pathway, which is a critical regulator of tumor development, progression, and resistance to therapy (17). PIK3CG plays a role in influencing cancer progression and the tumor microenvironment, particularly through immune cell signaling. PIK3CG (PI3Kgamma), a member of the PI3K enzyme family, is directly regulated by Gβγ subunits and Ras within the G protein-coupled receptor (GPCR) pathway. It plays a key role in regulating cellular inflammation and immune responses (73). PIK3CG is overexpressed in numerous invasive human breast tumors, and its expression levels are associated with the metastatic potential of breast cancer cell lines (74). Previous research suggests that PIK3CG may enhance breast cancer cell migration and invasion while preventing anoikis (74). Additionally, Zhang et al. proposed that PIK3CG contributes to breast cancer aggressiveness by not only facilitating cell migration but also sustaining cancer stemness (75).

PIK3R1, a regulatory subunit of PI3K, is involved in controlling pathway activity, and its mutations can lead to hyperactivation, promoting tumor progression (76). mTOR, a central regulator of cell growth and survival, is often dysregulated in breast cancer, with overactivation linked to cell proliferation and therapeutic resistance (77). PIK3R1 encodes the regulatory subunit (p85α) of the PI3K complex, and multiple studies have shown its expression is often reduced in human cancers (78), contributing to tumor development (76). In invasive ductal carcinoma of the breast, PIK3R1 is among the genes identified as differentially expressed when compared to normal tissue (79). Cizkova et al. further demonstrated that reduced p85 expression serves as an independent negative prognostic marker in breast cancer (78). Collectively, these findings indicate that PIK3R1 functions as a tumor suppressor (76). Our findings suggest that PIK3R1 could potentially serve as a clinically valuable independent prognostic marker in breast cancer. Furthermore, reduced PIK3R1 expression, along with PIK3CA mutations, may indicate a favorable response to therapies targeting the PI3K pathway, such as PI3K inhibitors or inhibitors of downstream signaling molecules like mTOR inhibitors (80, 81). PIK3CA, one of the most commonly mutated genes in breast cancer, especially in HR+ and HER2+ subtypes, drives hyperactivation of the PI3K/AKT/mTOR pathway, making it a key target for PI3K inhibitors (73, 82, 83). Therefore, it seems that PIK3R1 underexpression and PIK3CA mutations could be investigated as potential predictors of resistance to ERBB2 inhibitors, including trastuzumab (84).

In addition, PIK3CD, although primarily involved in immune regulation, also contributes to tumor progression through its effects on the tumor microenvironment, representing a potential target for immunotherapies (22, 85).

The last of the discussed differentiating genes is *mTOR*, whose overexpression we demonstrated in breast cancer samples compared to the control. Studies have shown that mTOR is frequently overexpressed in breast cancer tissues compared to normal breast tissues. The high expression of mTOR has been linked to increased tumor cell proliferation and a more aggressive cancer phenotype. This overexpression is often associated with poor prognosis, as it correlates with faster disease progression, resistance to therapies, and higher tumor grade (86, 87).

In a study by Mutee et al., breast cancer tissues exhibited a significantly higher expression of the mTOR protein across varying intensities compared to normal breast tissues. However, no significant associations were observed between mTOR protein expression and clinicopathological factors such as age group, clinical stage, or receptor status (based on 78 breast cancer and 53 normal tissue samples) (88). Similarly, Cheng et al. found that 54.9% of breast cancer cases (out of 71 samples) were mTOR-positive, with significantly higher expression compared to 21.9% of normal tissues (from 32 samples) (89). Together, these genes highlight the importance of the PI3K/AKT/mTOR pathway in breast cancer biology and treatment strategies (90).

Our study highlights the elevated expression of specific proteins, such as COL4A1 and MAPK3, in TNBC, which contributes to its aggressive clinical behavior. COL4A1, is implicated in ECM remodeling and tumor microenvironment alterations (91). The increased concentration of COL4A1 in TNBC suggests its role in enhancing tumor cell invasion and migration by providing structural support and activating signaling pathways that promote metastasis (92). Moreover, overexpression of COL4A1 has been linked to angiogenesis, facilitating the formation of new blood vessels that support rapid tumor growth and dissemination (93). These findings align with the highly invasive and metastatic characteristics of TNBC, underscoring the therapeutic potential of targeting COL4A1 to mitigate ECM-driven tumor progression.

Similarly, MAPK3, a key player in the MAPK signaling pathway, is significantly elevated in TNBC. MAPK3 regulates processes such as cell proliferation, survival, and differentiation, which are often dysregulated in aggressive cancers (94). The heightened activity of MAPK3 in TNBC may drive the rapid growth and survival of tumor cells, particularly in environments with limited resources or high stress. This overexpression supports the aggressive phenotype of TNBC, suggesting that targeting the MAPK signaling pathway could be a viable strategy to inhibit tumor growth and metastasis in this challenging subtype (95).

In addition to these proteins, our findings indicate increased levels of PIK3CG and mTOR, highlighting their potential as therapeutic targets. PIK3CG, a catalytic subunit of the PI3K enzyme, plays a central role in the PI3K/AKT/mTOR pathway, which regulates cell growth, metabolism, and survival. Overexpression of PIK3CG in TNBC suggests its involvement in enhancing cancer cell proliferation and resistance to apoptosis, contributing to tumor aggressiveness. Targeting PIK3CG with specific inhibitors, in combination with other pathway modulators, could effectively disrupt these processes and limit tumor progression (73, 96, 97).

Similarly, elevated mTOR levels in TNBC reinforce the pathway’s central role in promoting cellular metabolism and growth under nutrient-scarce conditions, which are common in the tumor microenvironment. mTOR is a downstream effector in the PI3K/AKT pathway and a critical regulator of autophagy, protein synthesis, and angiogenesis (17). Its upregulation in TNBC highlights its importance in maintaining the energy demands of rapidly dividing tumor cells. Combining mTOR inhibitors with agents targeting upstream regulators, such as PIK3CG, may provide synergistic effects, offering a robust approach to addressing TNBC’s aggressive nature (98, 99).

These findings suggest that the combined targeting of pathways involving COL4A1, MAPK3, PIK3CG, and mTOR could offer significant therapeutic benefits. Such combination therapies may effectively counteract the compensatory mechanisms often observed with single-agent treatments, potentially improving outcomes for patients with TNBC.

The STRING network analysis highlights the critical interactions among key molecular players, including MAPK3, PIK3CA, and mTOR, which serve as central hubs in the PI3K/AKT/mTOR pathway. The dense connectivity and high interaction scores within this network emphasize the significant roles these nodes play in oncogenic signaling. Notably, the observed crosstalk between the MAPK and PI3K/AKT/mTOR pathways suggests potential synergistic effects of targeting multiple nodes within these signaling cascades. MAPK3’s involvement in cell proliferation and survival, alongside PIK3CA and mTOR’s roles in metabolic regulation and tumor progression, underlines their contribution to breast cancer pathophysiology, particularly in aggressive subtypes like TNBC and HER2-positive cancers.

In our study, Kaplan-Meier survival analysis also revealed that for luminal A, poor OS was associated with high PIK3CA expression, alongside low COL1A1, MAPK3, and PIK3R1 activity. In luminal B HER2- cancers, only high COL4A1 expression significantly impacted OS. For non-luminal HER2+, a combination of high COL4A1 and low PIK3CD and PIK3CG activity was linked to worse OS. In TNBC, poor survival was associated with high COL4A1 and PIK3R1 expression, as well as low MAPK3, PIK3CD, and PIK3CG activity. This highlights the multifaceted involvement of ECM components, PI3K isoforms, and MAPK signaling in the cancer progression, especially in more aggressive subtypes. The co-targeting of MAPK and PI3K/AKT pathways has gained traction as a promising therapeutic strategy. Such strategy could disrupt the compensatory mechanisms often observed in single pathway targeting, thereby enhancing therapeutic efficacy and mitigating resistance. Luminal A breast cancer often harbors PIK3CA mutations, making the PI3K pathway a key target for therapeutic intervention. The PI3K inhibitor alpelisib has demonstrated significant benefits when combined with fulvestrant, showing improved progression-free survival (PFS) in clinical trials (100). Similarly, the combination of the next-generation PI3K inhibitor inavolisib with palbociclib and fulvestrant has also led to longer PFS, indicating a promising approach for treating luminal breast cancer (101). In addition to targeting the PI3K pathway, ECM remodeling and the overexpression of components like collagen IV have gained attention as potential therapeutic strategies. Fatherree et al. emphasize that targeting collagen IV, along with other ECM components, may offer new strategies to treat cancers where ECM remodeling plays a critical role (102). Non-luminal HER2+ and TNBC subtypes are associated with more aggressive behavior, highlighting the need for therapeutic approaches that address both molecular pathways and ECM remodeling (103). PIK3R1 plays a crucial role in the progression of TNBC and could serve as an important therapeutic target, as highlighted by Cobleight et al. (104). Although there are ongoing clinical trials investigating the efficacy of PI3K inhibitors in breast cancer, no specific trials are currently focused solely on targeting PIK3R1 in TNBC Our findings, supported by both STRING network insights and differential expression profiles, highlight the potential of targeting the PI3K/AKT/mTOR pathways in breast cancer subtypes.

In our analysis, we also demonstrated that the expression of selected genes could be regulated by miRNA molecules. For all the miRNAs identified in the microarray experiment, predictive analysis indicated interactions with the selected mRNAs, and we observed decreased expression in cancer tissues compared to controls.

We showed that miR-190a-3p, a key regulator of gene expression and pathways important in breast cancer [88], is potentially involved in the regulation of *COL4A1*, *MAPK3*, *PIK3CG*, and *PIK3R1*, all of which are integral to pathways associated with breast cancer progression. Jiang et al. demonstrated that hypoxia leads to the silencing of miR-190a-3p expression, contributing to uncontrolled cell proliferation (105). Notably, Jin et al. found that overexpression of miR-190a-3p results in the inactivation of the PI3K/AKT signaling pathway in glioma (106), suggesting that the silencing of this miRNA observed in our breast cancer tissue samples compared to controls could contribute to increased expression of genes encoding proteins of the PI3K/AKT pathway, enhancing the metastatic potential of cancer cells.

The predictive analysis also indicated a potential interaction between mTOR and miR-4729. Liu et al. found that reduced expression of miR-4729 leads to increased endothelial cell proliferation and enhanced angiogenesis (107). The downregulation of miR-4729 observed in our study supports its role in promoting angiogenesis and tumor growth in breast cancer (108). Another key miRNA was miR-19a-3p. In breast cancer, miR-19a-3p has been identified as a key regulator of *PIK3CA*, a central component of the PI3K/AKT pathway, which is known to promote tumor growth and survival. Wa et al. demonstrated that reduced expression of miR-19a-3p enhances cancer cell invasion and migration by inhibiting TGF-β signaling (109). Furthermore, overexpression of miR-19a-3p has been linked to increased metastatic potential in several cancer types, including colorectal and lung cancers (110, 111). However, in breast cancer and non-melanoma skin cancers, miR-19a-3p often exhibits reduced expression, suggesting a tumor-suppressive role in certain contexts (112, 113). These context-dependent roles highlight its complex contribution to breast cancer progression and underscore its potential as a therapeutic target. In addition to its role in metastasis, miR-19a-3p has been implicated in drug resistance. Its ability to modulate PI3K/AKT and TGF-β pathways suggests that dysregulation of miR-19a-3p could contribute to resistance mechanisms, particularly in therapies targeting these pathways. These findings support further exploration of miR-19a-3p as a biomarker for predicting metastatic potential and therapeutic response in breast cancer.

The final differentiating miRNA was miR-30d, which is being considered as a complementary molecular marker for the diagnosis and treatment efficacy assessment in breast cancer patients (114). Observations by Ma et al. indicate that reduced expression of miR-30d-5p in TNBC tissue promotes EMT by downregulating key inhibitors of mesenchymal transition (115). This loss of miR-30d-5p function allows for increased migration and invasion of cancer cells, exacerbating tumor progression. Furthermore, miR-30d-5p has been linked to drug resistance in multiple cancer models, as its suppression has been associated with poor response to chemotherapeutic agents (99, 116). In breast cancer, miR-30d-5p’s ability to regulate the PI3K/AKT pathway provides a possible mechanism for overcoming resistance to targeted therapies. By reinstating miR-30d-5p expression, it may be possible to suppress EMT, reduce metastatic potential, and improve therapeutic efficacy. These findings position miR-30d-5p as a promising molecular target for interventions aimed at controlling metastasis and drug resistance in aggressive breast cancer subtypes.

This study has several limitations that warrant discussion. One of the primary limitations is the lack of experimental functional validation for the bioinformatically predicted miRNA-mRNA interactions. While computational tools and databases were rigorously used to identify potential miRNA targets and regulatory pathways, the functional roles of these interactions were not directly validated in this research. Future studies will address this by employing experimental approaches, such as luciferase reporter assays, RNA immunoprecipitation, or CRISPR-based techniques, to confirm the regulatory mechanisms of the identified miRNAs on their target genes. This step is crucial to solidify the biological relevance of the miRNA-mRNA interactions highlighted in our findings. Despite this limitation, the broader implications of the study are significant. The results underscore the potential of molecular profiling in advancing personalized treatment strategies for breast cancer patients. Specifically, the identification of miRNAs such as miR-129, miR-19a-3p, and miR-30d-5p, as well as proteins like COL4A1, MAPK3, PIK3CG, and mTOR, provides valuable insights into the molecular mechanisms underpinning aggressive breast cancer subtypes, such as TNBC. Understanding the regulatory networks that drive tumor progression and metastasis could inform the development of targeted therapeutic approaches. Furthermore, integrating miRNA and protein expression data into clinical workflows has the potential to refine prognostic tools and guide personalized treatment decisions. For instance, targeting the PI3K/AKT/mTOR and MAPK pathways in combination therapies could offer more effective treatment options for TNBC, a subtype with limited therapeutic avenues. The translational potential of these findings lies in bridging molecular discoveries with actionable clinical strategies, ultimately improving outcomes for breast cancer patients. Future research and well-designed clinical trials are warranted to rigorously investigate these pathways and validate the translational potential of our findings, facilitating their integration into clinically actionable strategies for breast cancer management.

This study offers valuable insights into the distinct mRNA expression profiles and miRNA regulators of the PI3K/AKT/mTOR signaling pathway across various breast cancer subtypes. Key genes, including *COL1A1*, *COL4A1*, *MAPK3*, *PIK3CA*, *PIK3R1*, and *mTOR*, were identified as differentially expressed and potentially regulated by miRNAs such as miR-190a-3p, miR-4729, and miR-19a-3p. These genes play critical roles in tumor initiation, progression, and metastasis, particularly in aggressive subtypes like TNBC. The findings suggest that targeting specific components of the PI3K/AKT/mTOR pathway and their miRNA regulators could provide new opportunities for personalized therapeutic interventions. Future research should prioritize the functional validation of these regulatory networks and investigate their clinical utility as biomarkers or therapeutic targets.

## Data Availability

The original contributions presented in the study are included in the article, further inquiries.

## Glossary

AKT: Protein Kinase B
BMI: Body Mass Index
CAF: Cancer-Associated Fibroblasts
CI: Confidence Interval
COL1A1: Collagen Type I Alpha 1 Chain
COL1A2: Collagen Type I Alpha 2 Chain
COL2A1: Collagen Type II Alpha 1 Chain
COL4A1: Collagen Type IV Alpha 1 Chain
COL4A4: Collagen Type IV Alpha 4 Chain
COL4A6: Collagen Type IV Alpha 6 Chain
COL6A2: Collagen Type VI Alpha 2 Chain
ECM: Extracellular Matrix
ELISA: Enzyme-Linked Immunosorbent Assay
FC: Fold Change
FDR: False Discovery Rate
HER2: Human Epidermal Growth Factor Receptor 2
HR: Hazard Ratio
KEGG: Kyoto Encyclopedia of Genes and Genomes
MAP2K2: Mitogen-Activated Protein Kinase Kinase 2
MAPK: Mitogen-Activated Protein Kinase
MAPK1: Mitogen-Activated Protein Kinase 1
MAPK3: Mitogen-Activated Protein Kinase 3
miRNA: MicroRNA
mTOR: Mammalian Target of Rapamycin
OS: Overall Survival
PI3K: Phosphoinositide 3-Kinase
PIK3CA: Phosphatidylinositol-4,5-Bisphosphate 3-Kinase Catalytic Subunit Alpha
PIK3CB: Phosphatidylinositol-4,5-Bisphosphate 3-Kinase Catalytic Subunit Beta
PIK3CD: Phosphatidylinositol-4,5-Bisphosphate 3-Kinase Catalytic Subunit Delta
PIK3CG: Phosphatidylinositol-4,5-Bisphosphate 3-Kinase Catalytic Subunit Gamma
PIK3R1: Phosphatidylinositol-3-Kinase Regulatory Subunit Alpha
PIK3R4: Phosphatidylinositol-3-Kinase Regulatory Subunit 4
qRT-PCR: Quantitative Reverse Transcription Polymerase Chain Reaction
STRING: Search Tool for the Retrieval of Interacting Genes/Proteins
T1N0M0: Tumor, Node, and Metastasis Classification
TNBC: Triple-Negative Breast Cancer.
