# Corrigendum: Distinct mRNA expression profiles and miRNA regulators of the PI3K/AKT/mTOR pathway in breast cancer: insights into tumor progression and therapeutic targets

**DOI:** 10.3389/fonc.2025.1558771

**Published:** 2025-04-08

**Authors:** Tomasz Sirek, Katarzyna Król-Jatręga, Przemysław Borawski, Nikola Zmarzły, Dariusz Boroń, Piotr Ossowski, Olga Nowotny-Czupryna, Kacper Boroń, Dominika Janiszewska-Bil, Elżbieta Mitka-Krysiak, Beniamin Oskar Grabarek

**Affiliations:** ^1^ Department of Plastic Surgery, Faculty of Medicine, Academia of Silesia, Katowice, Poland; ^2^ Department of Plastic and Reconstructive Surgery, Hospital for Minimally Invasive and Reconstructive Surgery in Bielsko-Biała, Bielsko-Biala, Poland; ^3^ Independent Researcher, Włocławek, Poland; ^4^ Department of Medical and Health Sciences, Collegium Medicum, WSB University, Dabrowa Górnicza, Poland; ^5^ Department of Gynecology and Obstetrics with Gynecologic Oncology, Ludwik Rydygier Memorial Specialized Hospital, Kraków, Poland; ^6^ Department of Gynecology and Obstetrics, TOMMED Specjalisci od Zdrowia, Katowice, Poland; ^7^ University of Economics and Humanities in Warsaw, Warszawa, Poland; ^8^ Department of Molecular, Biology Gyncentrum Fertility Clinic, Katowice, Poland

**Keywords:** breast cancer, miRNA, PI3K/Akt/mTOR pathway, molecular marker, mRNA

In the published article, there was an error in **Figure 1** as published. The incorrect image was displayed. The corrected **Figure 1** and its caption “Venn diagram of genes differentiating breast cancer from the control. LumA, luminal A; LumB, luminal B; HER2, human epidermal growth factor receptor 2; TNBC, triple-negative breast cancer; C, control; *COL1A1*, collagen type I alpha 1; COL1A2, collagen type I alpha 2; *COL2A1*, Collagen Type II Alpha 1 Chain; *COL4A1*, Collagen Type IV Alpha 1 Chain; *COL4A4*, Collagen Type IV Alpha 4 Chain; *COL4A6*, Collagen Type IV Alpha 6 Chain; *COL6A2*, Collagen Type VI Alpha 2 Chain; *PIK3CA*, Phosphatidylinositol-4,5-Bisphosphate 3-Kinase Catalytic Subunit Alpha; *PIK3C*B, Phosphatidylinositol-4,5-Bisphosphate 3-Kinase Catalytic Subunit Beta; *PIK3CD*, Phosphatidylinositol-4,5-Bisphosphate 3-Kinase Catalytic Subunit Delta; *PIK3CG*, Phosphatidylinositol-4,5-Bisphosphate 3-Kinase Catalytic Subunit Gamma; *PIK3R1*, Phosphoinositide-3-Kinase Regulatory Subunit 1; *PIK3R4*, Phosphoinositide-3-Kinase Regulatory Subunit 4; *MAPK1*, Mitogen-Activated Protein Kinase 1; *MAPK3*, Mitogen-Activated Protein Kinase 3; *MAP2K2*, Mitogen-Activated Protein Kinase Kinase 2; *mTOR*, Mechanistic Target of Rapamycin” appear below.

**Figure 1:**
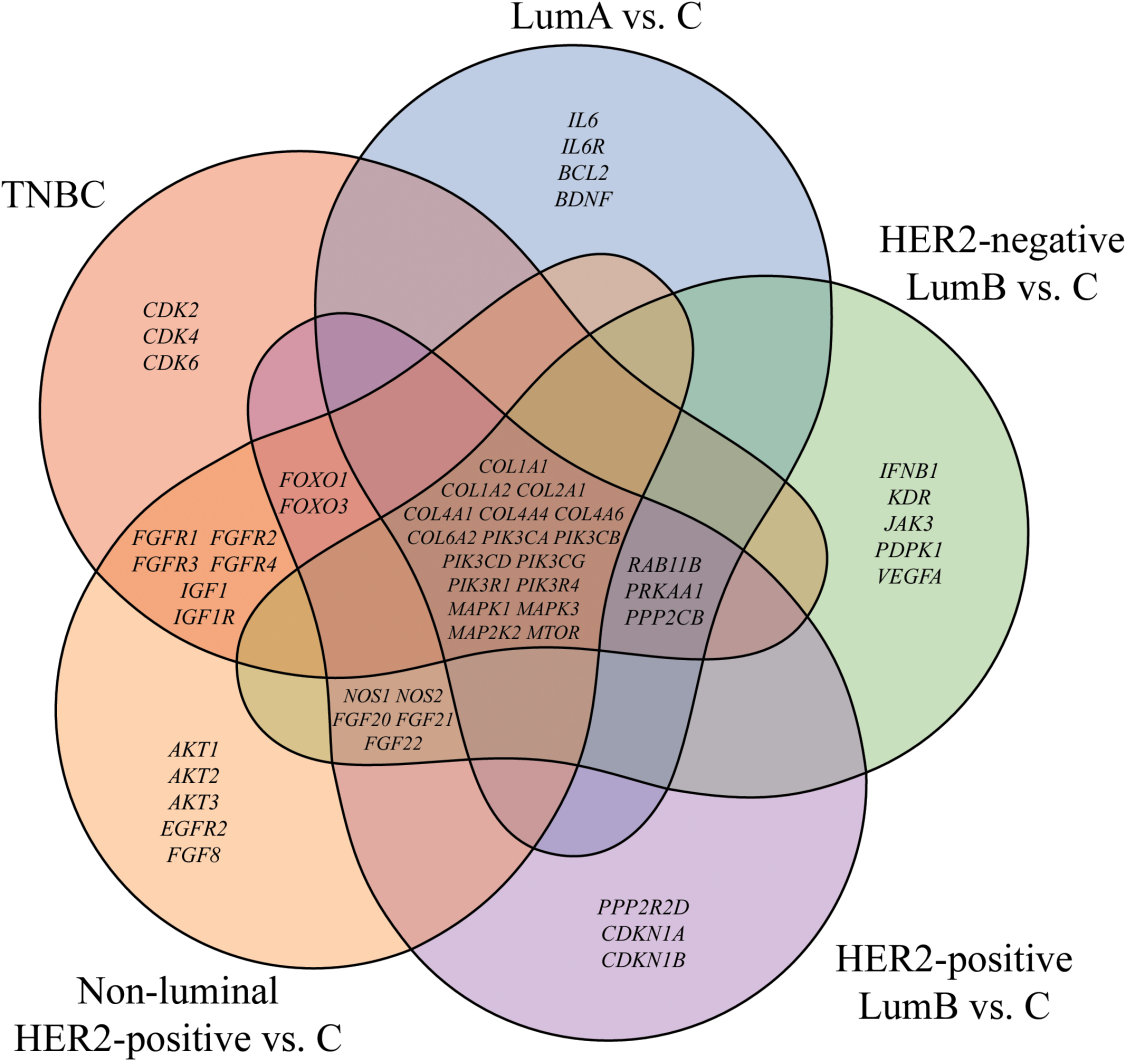
Venn diagram of genes differentiating breast cancer from the control. LumA, luminal A; LumB, luminal B; HER2, human epidermal growth factor receptor 2; TNBC, triple-negative breast cancer; C, control; *COL1A1*, collagen type I alpha 1; COL1A2, collagen type I alpha 2; *COL2A1*, Collagen Type II Alpha 1 Chain; *COL4A1*, Collagen Type IV Alpha 1 Chain; *COL4A4*, Collagen Type IV Alpha 4 Chain; *COL4A6*, Collagen Type IV Alpha 6 Chain; *COL6A2*, Collagen Type VI Alpha 2 Chain; *PIK3CA*, Phosphatidylinositol-4,5-Bisphosphate 3-Kinase Catalytic Subunit Alpha; *PIK3C*B, Phosphatidylinositol-4,5-Bisphosphate 3-Kinase Catalytic Subunit Beta; *PIK3CD*, Phosphatidylinositol-4,5-Bisphosphate 3-Kinase Catalytic Subunit Delta; *PIK3CG*, Phosphatidylinositol-4,5-Bisphosphate 3-Kinase Catalytic Subunit Gamma; *PIK3R1*, Phosphoinositide-3-Kinase Regulatory Subunit 1; *PIK3R4*, Phosphoinositide-3-Kinase Regulatory Subunit 4; *MAPK1*, Mitogen-Activated Protein Kinase 1; *MAPK3*, Mitogen-Activated Protein Kinase 3; *MAP2K2*, Mitogen-Activated Protein Kinase Kinase 2; *mTOR*, Mechanistic Target of Rapamycin.

In the published article, there was an error in **Figure 2** as published. The incorrect image was displayed. The corrected **Figure 2** and its caption “Expression profile of selected genes determined by qRT-PCR. LumA, luminal A; LumB, luminal B; HER2, human epidermal growth factor receptor 2; TNBC, triple-negative breast cancer; C, control; COL1A1, collagen type I alpha 1; COL1A2, collagen type I alpha 2; *COL2A1*, Collagen Type II Alpha 1 Chain; *COL4A1*, Collagen Type IV Alpha 1 Chain; *COL4A4*, Collagen Type IV Alpha 4 Chain; *COL4A6*, Collagen Type IV Alpha 6 Chain; *COL6A2*, Collagen Type VI Alpha 2 Chain; *PIK3CA*, Phosphatidylinositol-4,5-Bisphosphate 3-Kinase Catalytic Subunit Alpha; *PIK3CB*, Phosphatidylinositol-4,5-Bisphosphate 3-Kinase Catalytic Subunit Beta; *PIK3CD*, Phosphatidylinositol-4,5-Bisphosphate 3-Kinase Catalytic Subunit Delta; *PIK3CG*, Phosphatidylinositol-4,5-Bisphosphate 3-Kinase Catalytic Subunit Gamma; *PIK3R1*, Phosphoinositide-3-Kinase Regulatory Subunit 1; *PIK3R4*, Phosphoinositide-3-Kinase Regulatory Subunit 4; *MAPK1*, Mitogen-Activated Protein Kinase 1; *MAPK3*, Mitogen-Activated Protein Kinase 3; *MAP2K2*, Mitogen-Activated Protein Kinase Kinase 2; *mTOR*, Mechanistic Target of Rapamycin” appear below.

**Figure 2:**
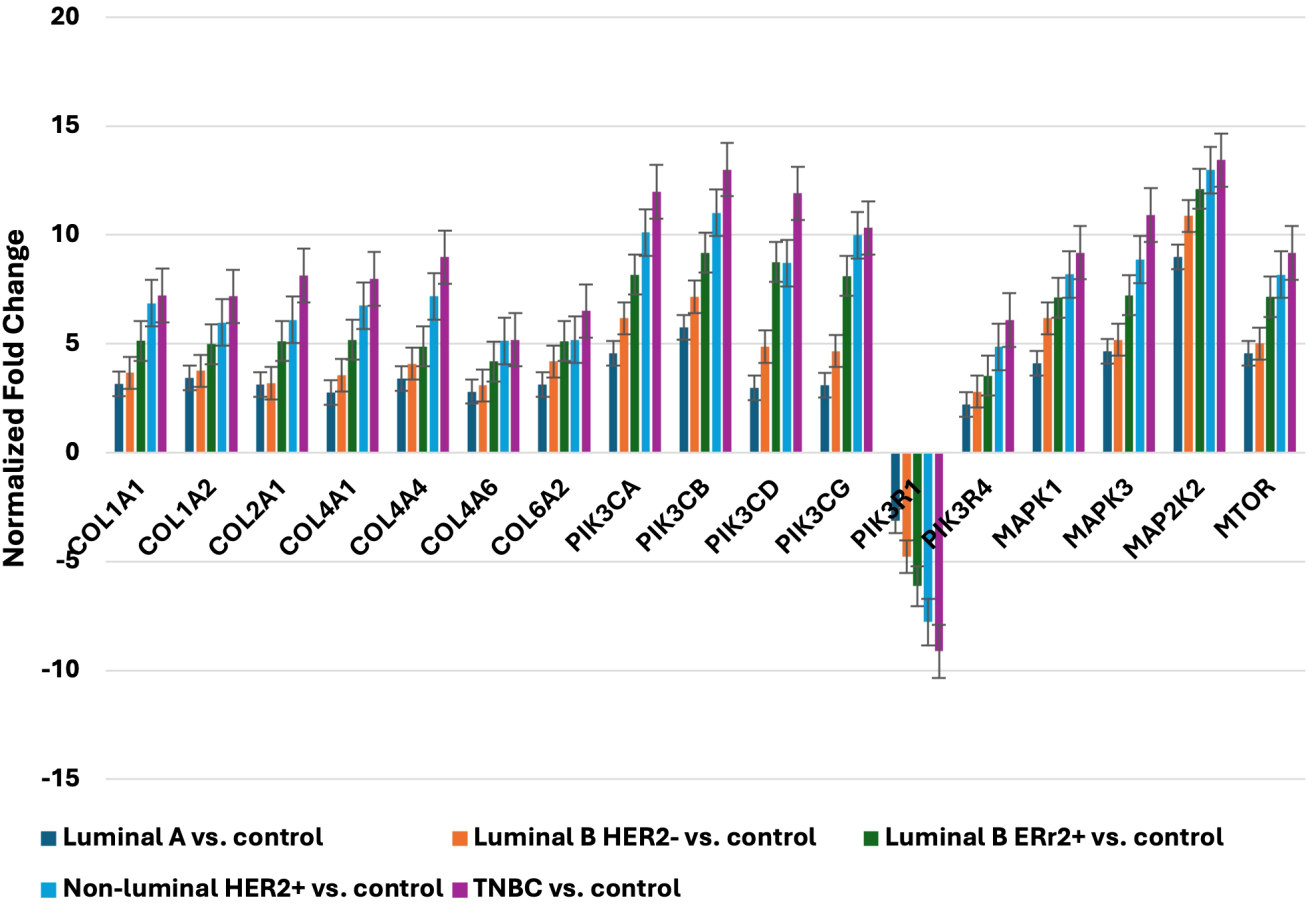
Expression profile of selected genes determined by qRT-PCR. LumA, luminal A; LumB, luminal B; HER2, human epidermal growth factor receptor 2; TNBC, triple-negative breast cancer; C, control; *COL1A1*, collagen type I alpha 1; *COL1A2*, collagen type I alpha 2; *COL2A1*, Collagen Type II Alpha 1 Chain; *COL4A1*, Collagen Type IV Alpha 1 Chain; *COL4A4*, Collagen Type IV Alpha 4 Chain; *COL4A6*, Collagen Type IV Alpha 6 Chain; *COL6A2*, Collagen Type VI Alpha 2 Chain; *PIK3CA*, Phosphatidylinositol-4,5-Bisphosphate 3-Kinase Catalytic Subunit Alpha; *PIK3CB*, Phosphatidylinositol-4,5-Bisphosphate 3-Kinase Catalytic Subunit Beta; *PIK3CD*, Phosphatidylinositol-4,5-Bisphosphate 3-Kinase Catalytic Subunit Delta; *PIK3CG*, Phosphatidylinositol-4,5-Bisphosphate 3-Kinase Catalytic Subunit Gamma; *PIK3R1*, Phosphoinositide-3-Kinase Regulatory Subunit 1; *PIK3R4*, Phosphoinositide-3-Kinase Regulatory Subunit 4; *MAPK1*, Mitogen-Activated Protein Kinase 1; *MAPK3*, Mitogen-Activated Protein Kinase 3; *MAP2K2*, Mitogen-Activated Protein Kinase Kinase 2; *mTOR*, Mechanistic Target of Rapamycin.

In the published article, there was an error in **Figure 3** as published. The incorrect image was displayed. The corrected **Figure 3** and its caption “Relationship network for the selected PI3K/AKT/mTOR pathway differentiation genes generated in the STRING database. COL1A1, collagen type I alpha 1; COL1A2, collagen type I alpha 2; COL2A1, Collagen Type II Alpha 1 Chain; COL4A1, Collagen Type IV Alpha 1 Chain; COL4A4, Collagen Type IV Alpha 4 Chain; COL4A6, Collagen Type IV Alpha 6 Chain; COL6A2, Collagen Type VI Alpha 2 Chain; PIK3CA, Phosphatidylinositol-4,5-Bisphosphate 3-Kinase Catalytic Subunit Alpha; PIK3CB, Phosphatidylinositol-4,5-Bisphosphate 3-Kinase Catalytic Subunit Beta; PIK3CD, Phosphatidylinositol-4,5-Bisphosphate 3-Kinase Catalytic Subunit Delta; PIK3CG, Phosphatidylinositol-4,5-Bisphosphate 3-Kinase Catalytic Subunit Gamma; PIK3R1, Phosphoinositide-3-Kinase Regulatory Subunit 1; PIK3R4, Phosphoinositide-3-Kinase Regulatory Subunit 4; MAPK1, Mitogen-Activated Protein Kinase 1; MAPK3, Mitogen-Activated Protein Kinase 3; MAP2K2, Mitogen-Activated Protein Kinase Kinase 2; mTOR, Mechanistic Target of Rapamycin” appear below.

**Figure 3:**
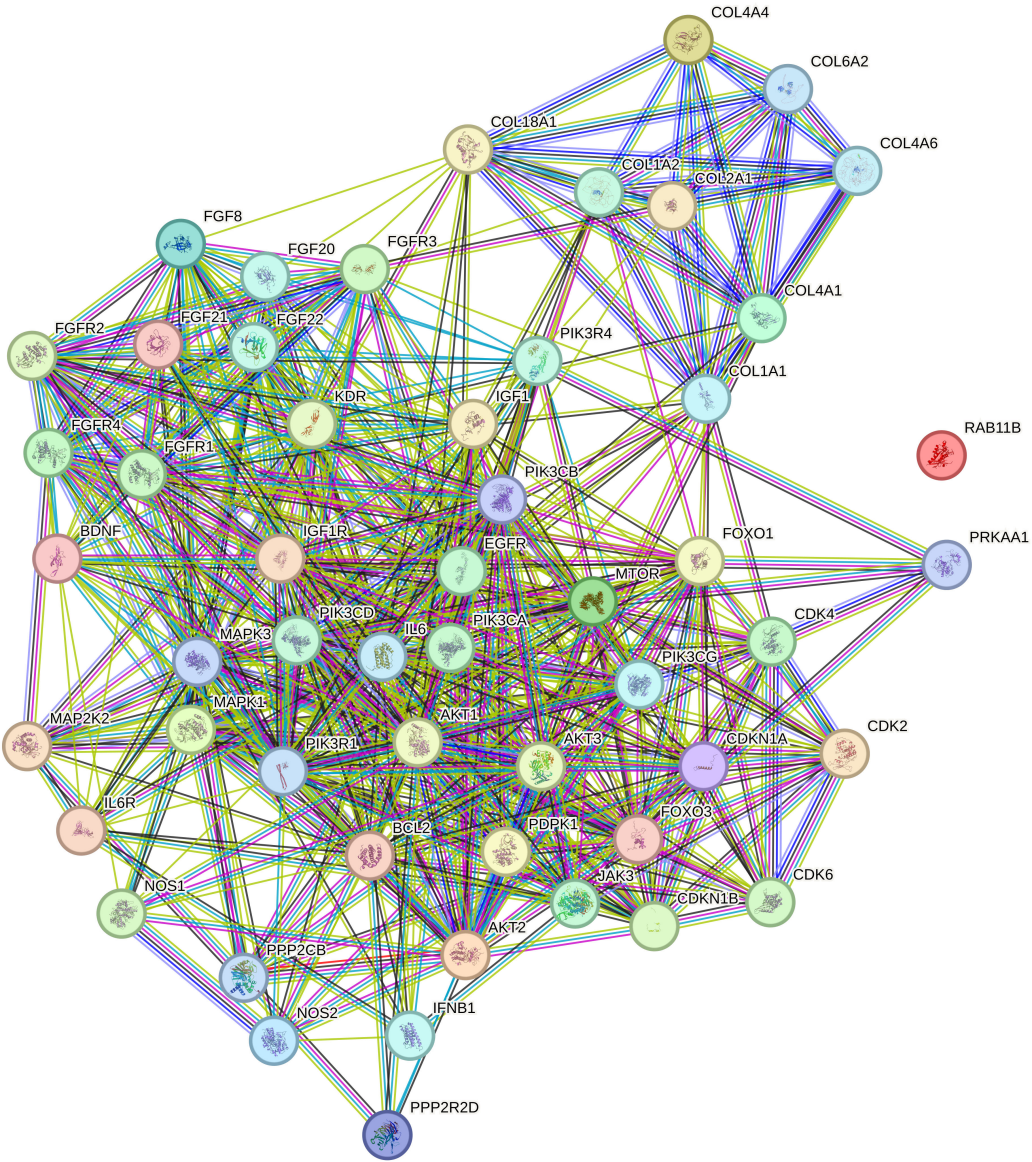
Relationship network for the selected PI3K/AKT/mTOR pathway differentiation genes generated in the STRING database. COL1A1, collagen type I alpha 1; COL1A2, collagen type I alpha 2; COL2A1, Collagen Type II Alpha 1 Chain; COL4A1, Collagen Type IV Alpha 1 Chain; COL4A4, Collagen Type IV Alpha 4 Chain; COL4A6, Collagen Type IV Alpha 6 Chain; COL6A2, Collagen Type VI Alpha 2 Chain; PIK3CA, Phosphatidylinositol-4,5-Bisphosphate 3-Kinase Catalytic Subunit Alpha; PIK3CB, Phosphatidylinositol-4,5-Bisphosphate 3-Kinase Catalytic Subunit Beta; PIK3CD, Phosphatidylinositol-4,5-Bisphosphate 3-Kinase Catalytic Subunit Delta; PIK3CG, Phosphatidylinositol-4,5-Bisphosphate 3-Kinase Catalytic Subunit Gamma; PIK3R1, Phosphoinositide-3-Kinase Regulatory Subunit 1; PIK3R4, Phosphoinositide-3-Kinase Regulatory Subunit 4; MAPK1, Mitogen-Activated Protein Kinase 1; MAPK3, Mitogen-Activated Protein Kinase 3; MAP2K2, Mitogen-Activated Protein Kinase Kinase 2; mTOR, Mechanistic Target of Rapamycin.

In the published article, there was an error in **Figure 6** as published. The incorrect image was displayed. The corrected **Figure 6** and its caption “Overall survival analysis for luminal B HER2+ subtype” appear below.

**Figure 6:**
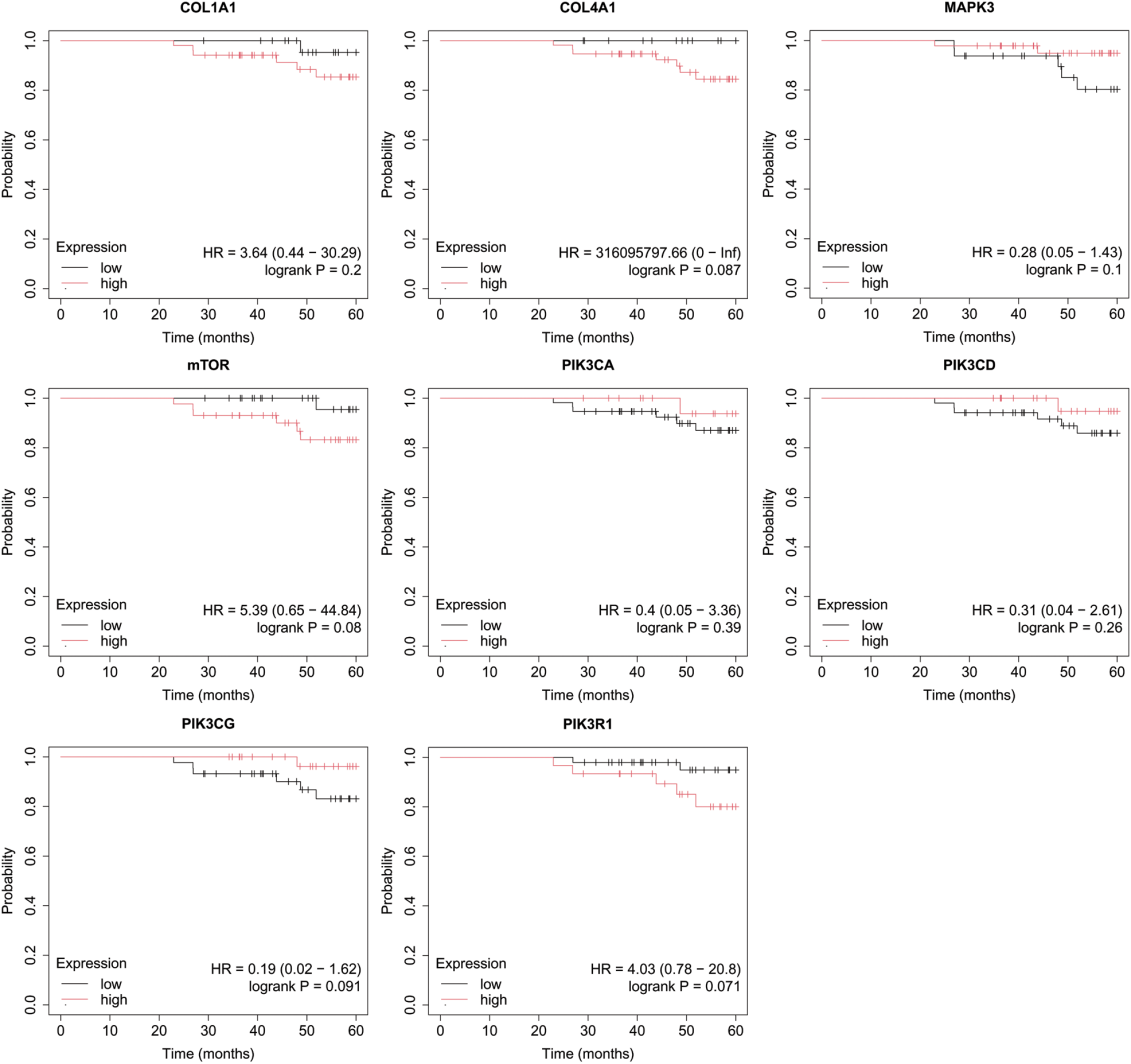
Overall survival analysis for luminal B HER2+ subtype.

In the published article, there was an error in **Figure 7** as published. The incorrect image was displayed. The corrected **Figure 7** and its caption “Overall survival analysis for non-luminal HER2+ cancers subtype” appear below.

**Figure 7:**
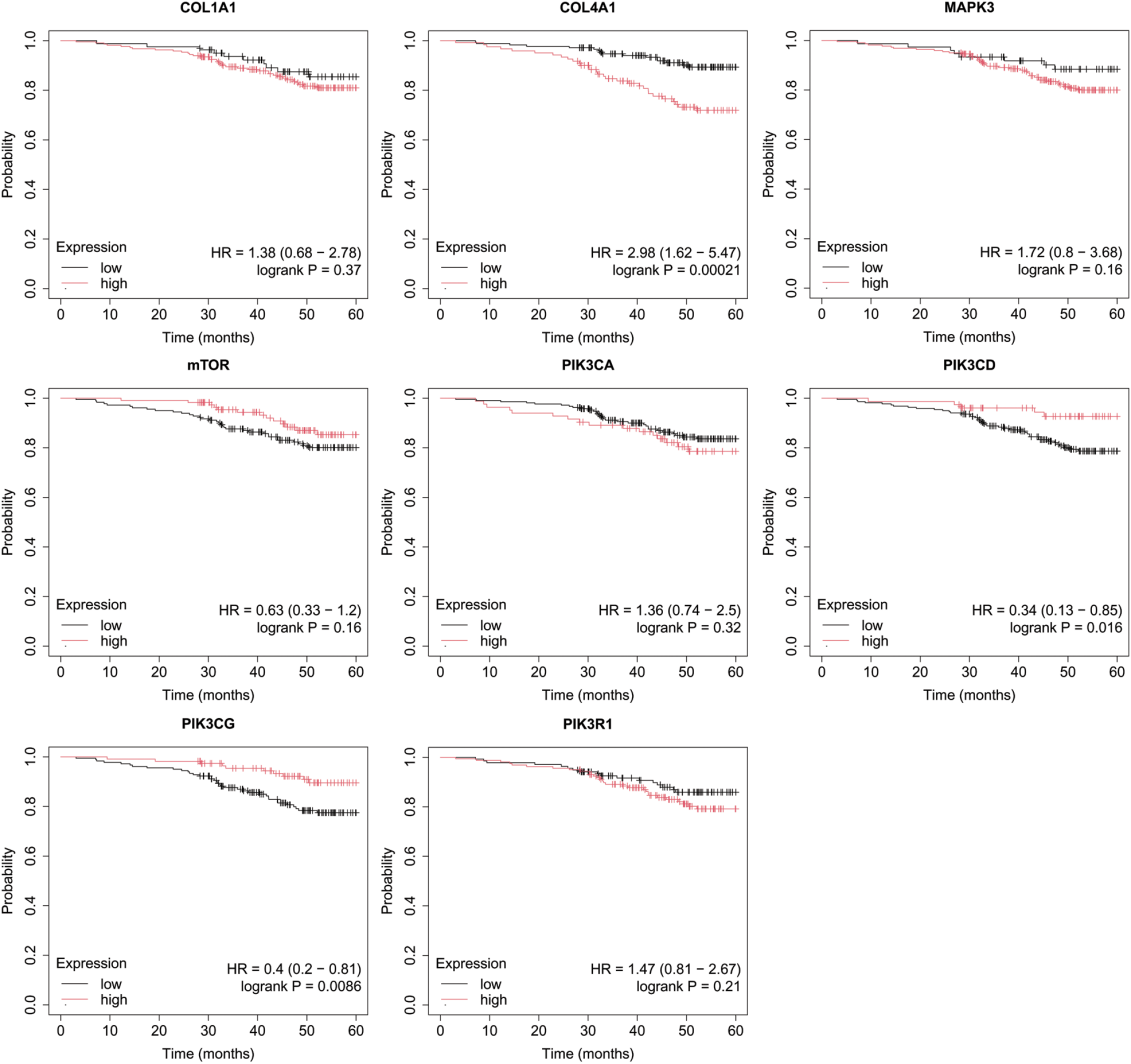
Overall survival analysis for non-luminal HER2+ cancers subtype.

In the published article, there was an error in **Figure 8** as published. The incorrect image was displayed. The corrected **Figure 8** and its caption “Overall survival analysis for TNBC subtype” appear below.

**Figure 8:**
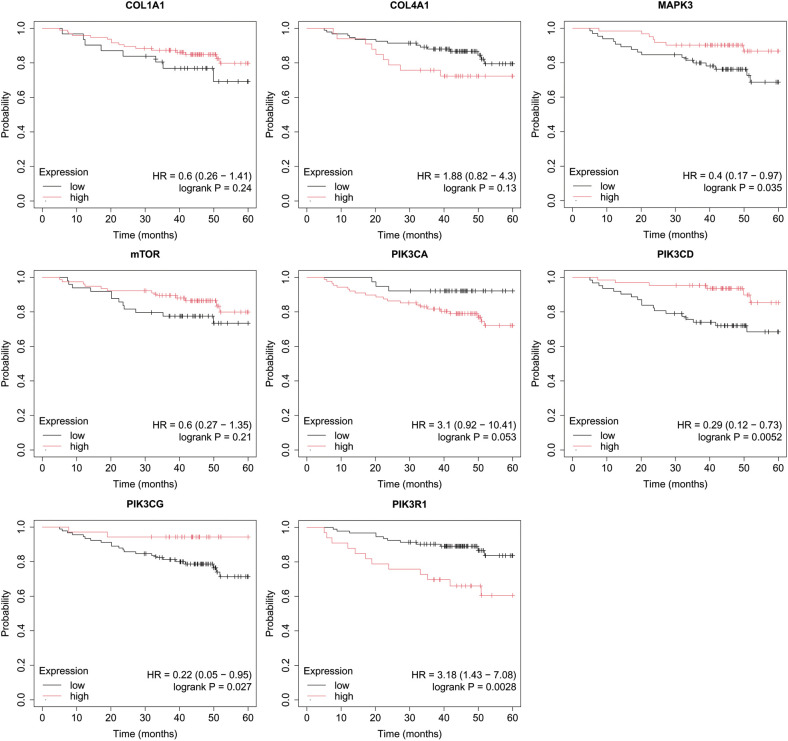
Overall survival analysis for TNBC subtype.

In the published article, there was an error. The title of sub-section 3.1 was incorrect.

A correction has been made to **Section 3. Results**, *Sub-section 3.1*. This sentence previously stated:

“Microarray profile of histaminergic system-related genes breast cancer samples in comparison with control tissue”

The corrected sentence appears below:

“Microarray profile of PI3K/AKT/mTOR pathway-related genes breast cancer samples in comparison with control tissue”

In the published article, there was an error. The title of sub-section 3.2 was incorrect.

A correction has been made to **Section 3. Results**, *Sub-section 3.2*. This sentence previously stated:

“Expression pattern of histaminergic system-related genes in breast cancer samples compared to control tissue analyzed by qRT-PCR”

The corrected sentence appears below:

“Expression pattern of PI3K/AKT/mTOR pathway-related genes in breast cancer samples compared to control tissue analyzed by qRT-PCR”

The authors apologize for these errors and state that this does not change the scientific conclusions of the article in any way. The original article has been updated.

